# Autophagy in health and disease: From molecular mechanisms to therapeutic target

**DOI:** 10.1002/mco2.150

**Published:** 2022-07-10

**Authors:** Guang Lu, Yu Wang, Yin Shi, Zhe Zhang, Canhua Huang, Weifeng He, Chuang Wang, Han‐Ming Shen

**Affiliations:** ^1^ Department of Physiology, Zhongshan School of Medicine Sun Yat‐sen University Guangzhou China; ^2^ State Key Laboratory of Biotherapy and Cancer Center, West China Hospital, and West China School of Basic Medical Sciences & Forensic Medicine Sichuan University and Collaborative Innovation Center for Biotherapy Chengdu China; ^3^ Department of Biochemistry Zhejiang University School of Medicine Hangzhou China; ^4^ State Key Laboratory of Trauma, Burn and Combined Injury, Institute of Burn Research Southwest Hospital Army Medical University Chongqing China; ^5^ Department of Pharmacology, Provincial Key Laboratory of Pathophysiology Ningbo University School of Medicine Ningbo Zhejiang China; ^6^ Department of Biomedical Sciences, Faculty of Health Sciences, Ministry of Education Frontiers Science Center for Precision Oncology University of Macau Macau China

**Keywords:** autophagy, cancer, cardiovascular diseases, metabolic diseases, neurodegenerative diseases, SARS‐CoV‐2

## Abstract

Macroautophagy/autophagy is an evolutionally conserved catabolic process in which cytosolic contents, such as aggregated proteins, dysfunctional organelle, or invading pathogens, are sequestered by the double‐membrane structure termed autophagosome and delivered to lysosome for degradation. Over the past two decades, autophagy has been extensively studied, from the molecular mechanisms, biological functions, implications in various human diseases, to development of autophagy‐related therapeutics. This review will focus on the latest development of autophagy research, covering molecular mechanisms in control of autophagosome biogenesis and autophagosome–lysosome fusion, and the upstream regulatory pathways including the AMPK and MTORC1 pathways. We will also provide a systematic discussion on the implication of autophagy in various human diseases, including cancer, neurodegenerative disorders (Alzheimer disease, Parkinson disease, Huntington's disease, and Amyotrophic lateral sclerosis), metabolic diseases (obesity and diabetes), viral infection especially SARS‐Cov‐2 and COVID‐19, cardiovascular diseases (cardiac ischemia/reperfusion and cardiomyopathy), and aging. Finally, we will also summarize the development of pharmacological agents that have therapeutic potential for clinical applications via targeting the autophagy pathway. It is believed that decades of hard work on autophagy research is eventually to bring real and tangible benefits for improvement of human health and control of human diseases.

## Graphical abstract

Macroautophagy/autophagy is an evolutionally conserved catabolic process induced under various stress conditions to maintain intracellular homeostasis. Dysregulation of autophagy is closely associated with various human diseases. This review will focus on the molecular mechanisms in control of autophagy, the implication of autophagy in various human diseases, and the development of pharmacological agents that target autophagy.

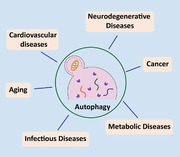

## 1 INTRODUCTION

Autophagy refers to a process in which the intracellular components such as abnormal proteins, damaged organelles, foreign pathogens, and other cellular components are degraded via lysosome. This catabolic process is evolutionarily conserved from yeast to mammalian cells. The history of autophagy research spans over 7 decades and marked with two Nobel prizes.^1^, ^2^ The modern concept of autophagy was coined in 1960s by Christian de Duve after he studied the function of lysosome, the key digestive organelle that he discovered in 1950s, which earned him the Nobel Prize in Physiology or Medicine in 1974. However, in the following 4 decades after the discovery of lysosome, the autophagy research remained a relatively small field, mainly due to limited research tools and lack of understanding of its molecular mechanisms. In 1990s, the ground‐breaking work by Yoshinori Ohsumi identified a series of autophagy‐related genes (Atgs, originally termed as Apgs) in control of autophagy using yeast as the model organism for studies.^3^ The discovery of Atgs and subsequent work on the molecular mechanisms elucidating the function of the proteins encoded by those Atgs earned Ohsumi the Nobel Prize in Physiology or Medicine in 2016. At present, a total of more than 40 Atgs have been identified and the autophagy field is still expanding, from the molecular mechanisms to biological functions and implications in health and disease.^4^, ^5^, ^6^

In mammalian cells, autophagy has been traditionally classified into the following three main types, macroautophagy, microautophagy, and chaperone‐mediated autophagy (CMA). Among them, macroautophagy is featured by the formation of a unique double‐membrane organelle, the autophagosome.^7^, ^8^ In contrast, both microautophagy and CMA bypass autophagosome formation and the cargos are directly delivered to lysosome.^9^, ^10^ At present, the majority of the autophagy research is on macroautophagy, or referred as autophagy hereafter in this review. On the other hand, depending on the nature of the cargos, autophagy can be categorized into general/nonselective and selective autophagy. For nonselective autophagy, the cellular cargos are engulfed into the autophagosomes randomly, a process usually induced by general stress conditions such as nutrient starvation.^11^ In contrast, selective autophagy refers to selective degradation of specific cargos, and so far, there are many types of selective autophagy being studied, such as mitophagy (selective degradation of mitochondria), endoplasmic reticulum (ER)‐phagy (selective degradation of ER), aggrephagy (selective degradation of protein aggregates), and xenophagy (selective degradation of invaded pathogens), just to name a few.^12^, ^13^, ^14^, ^15^ At present, it has been well studied that autophagy have important functions in various biological processes, such as cell survival and cell death, inflammation and immunity, development and differentiation, metabolic homeostasis, and so on. As such, autophagy is known to be closely implicated in the pathogenesis of human diseases.^16^, ^17^ In this review, we will mainly focus on nonselective macroautophagy to provide a systematic discussion on the latest development on the molecular mechanisms, the implication of autophagy in important human diseases including cancer, neurodegeneration, metabolic diseases, severe acute respiratory syndrome coronavirus 2 (SARS‐CoV‐2) infection, cardiovascular diseases, and aging. Moreover, we will also discuss the therapeutic potential of targeting autophagy in human diseases. Finally, we will highlight the challenges the autophagy research field is facing and the directions of future study.

## 2 MOLECULAR MECHANISMS IN CONTROL OF AUTOPHAGY

### 2.1 ATGs and autophagosome biogenesis

**FIGURE 1:**
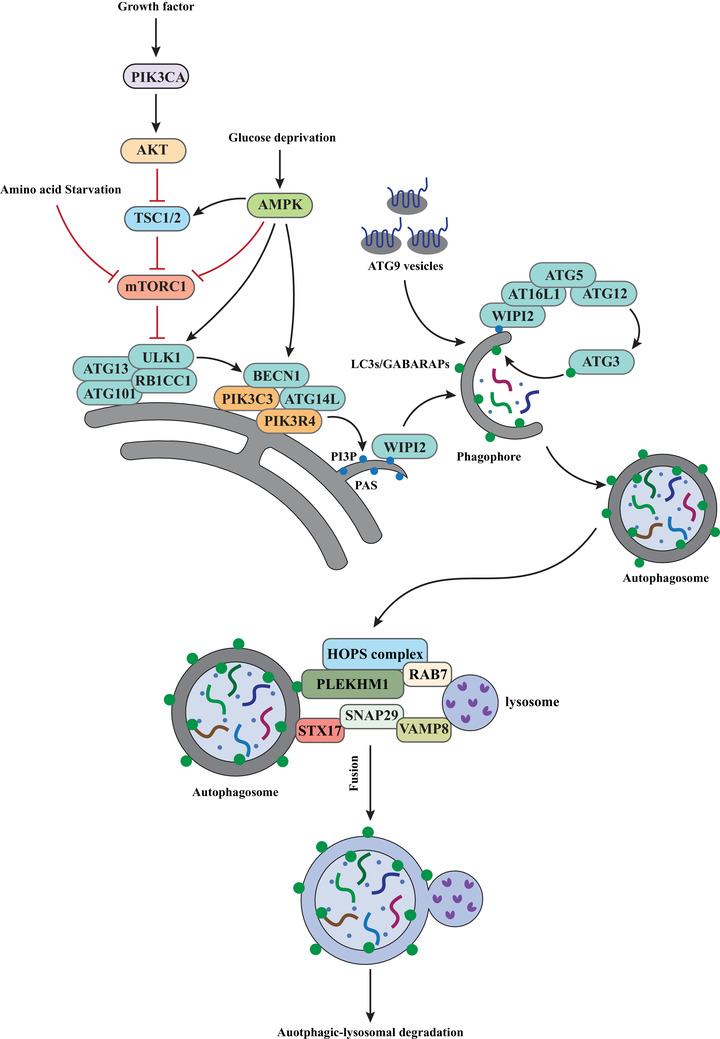
Molecular mechanisms in control of autophagy. Autophagy could be induced by various stress conditions such as amino starvation, glucose depletion, and others. The common target of these signaling pathways is the ULK1 complex. Under normal condition, MTORC1 inhibits ULK1 via phosphorylation. Under stress condition, MTORC1 is suppressed, leading to activation and recruitment of the ULK1 complex to PAS. ULK1 further activates PI3KC3–C1, resulting in generation of PtdIns3P (PI3P). WIPI2 then binds to PtdIns3P and further recruits ATG12–ATG5–ATG16L1 to the phagophore to mediate the lipidation of mATG8, which is essential for elongation and closure of the phagophore membrane. Additionally, the ATG9 vesicles are believe to supply membrane source, which also contributes to elongation of the phagophore membrane. Autophagosome–lysosome fusion is mediated by specific SNARE proteins, the HOPS tethering complex, small GTPases such as RAB7 and their effector PLEKHM1, and other factors detailed in the main text. The molecular mechanisms of these factors in autophagosome–lysosome fusion are discussed in the main text

The biogenesis of autophagosome is coordinated by the core ATG (autophagy related) proteins (Table 1). These ATG proteins form different complexes to drive the initiation, nucleation, expansion, and closure of autophagosome (Figure 1). Briefly, these complexes include: (i) the ULK1 complex, consisting of ULK1 (unc‐51 like autophagy activating kinase 1), RB1CC1/FIP200 (RB1 inducible coiled‐coil 1), ATG13 ang ATG101; (ii) the class III lipid kinase complex I (PI3KC3–C1), formed by PIK3C3/VPS34 (phosphatidylinositol 3‐kinase catalytic subunit type 3), PIK3R4/VPS15/p150 (phosphoinositide‐3‐kinase regulatory subunit 4), BECN1/Beclin 1, ATG14/ATG14L and NRBF2 (nuclear receptor binding factor 2); (iii) the WIPI (WD‐repeat protein interacting with phosphoinositides) family; (iv) the two ubiquitin‐like conjugation systems that facilitate conjugation of mATG8 to membrane‐resident phosphatidylethanolamine (PE); (v) the ATG9 vesicles.^45^, ^46^, ^47^

**TABLE 1:** Autophagy core machinery and their functions

Autophagy core machinery	Protein name	Functions	References
The ULK1 complex	ULK1/ATG1	Serine/threonine kinase; phosphorylates different components of the autophagy machinery to initiate autophagy.	^18^, ^19^
	ATG13	Interacts with ULK1; bridges ULK1 to RB1CC1.	^19^, ^20^, ^21^
	ATG101	Heterodimerizes with ATG13 via their HORMA domain to stabilize the binding of ATG13 to ULK1; enhances ULK1 activity.	^20^, ^22^
	RB1CC1/FIP200	Scaffold protein; possibly serves as scaffold for ULK1 and ATG13.	^21^, ^23^
The class III lipid kinase complex I (PI3KC3–C1)	PIK3C3/VPS34	PtdIns3 kinase; generates PtdIns3P.	^24^
	PIK3R4/VPS15	Scaffold and protein kinase; binds to PIK3C3 to form the catalytic arm of PI3KC3–C1.	^25^, ^26^
	BECN1	Forms the regulatory arm of PI3KC3–C1 via binding to ATG14.	^24^, ^27^
	ATG14	Directs PI3KC3–C1 to PAS.	^28^, ^29^
The WIPI family and WIPI–ATG2A complex	WIPI1	PtdIns3P binding protein.	^30^
	WIPI2	PtdIns3P binding protein; recruits ATG12–ATG5‐ATG16L1 complex to conjugate LC3/GABARAP to PE.	^30^, ^31^
	WIPI3	PtdIns3P binding protein; associates with TSC complex to regulate mTOR activity; complexes with ATG2A to regulate phagophore expansion.	^32^, ^33^
	WIPI4	PtdIns3P binding protein; complexes with ATG2A to regulate phagophore expansion.	^32^, ^33^, ^34^
	ATG2A	Complexes with WIPI3/WIPI4 to regulate phagophore expansion.	^33^, ^34^, ^35^
The two ubiquitin‐like conjugation systems	ATG4	Cysteine protease to cleave pro‐ATG8s; deconjugation of ATG8s from PE (phosphatidylethanolamine).	^36^, ^37^
	ATG7	E1‐like enzyme for both the ATG8s conjugation system and ATG12 conjugation system.	^4^, ^38^
	ATG3	E2‐like enzyme for the ATG8s conjugation system.	^4^, ^38^
	ATG10	E2‐like enzyme that conjugates ATG12 to ATG5.	^38^, ^39^
	ATG5	Conjugated by ATG12.	^38^, ^39^
	ATG12	The ubiquitin‐like molecule in the ATG12 conjugation system.	^38^, ^39^
	ATG16L1	Interacts with the ATG12–ATG5 conjugate to form the ATG12–ATG5‐ATG16L1 complex, which acts as the E3‐like enzyme to conjugate mATG8 to PE.	^40^
	mATG8	The ubiquitin‐like molecule in the ATG8s conjugation system.	^41^, ^42^
ATG9 vesicles	ATG9	Contributes membrane source to the forming phagophore.	^43^, ^44^, ^45^

#### 2.1.1 The ULK1 complex

Autophagy could be triggered under various stressed conditions such as amino acid starvation, glucose depletion, hypoxia, oxidative stress, and others. Upon autophagy induction, ULK1 is activated via autophosphorylation within its kinase domain at Thr180.^48^, ^49^, ^50^ ULK1 is a serine/threonine protein kinase that contains a protein kinase domain at its N‐terminus, while the C‐terminus is responsible for interaction with the C‐terminus of ATG13.^19^ ATG101 is fully composed of a HORMA (Hop1, Rev7, Mad2) domain and it heterodimerizes with ATG13 via the HORMA domain at its N terminus, which prevents the proteasomal degradation of ATG13 and strengthens the interaction between ATG13 and ULK1.^20^, ^22^ On the other hand, ATG13 also bridges ULK1 to the scaffolding subunit RB1CC1.^21^, ^23^ The assembly of the ULK1 complex is constitutive and not affected much by nutrient deprivation.^21^, ^51^ There is evidence indicating that the association of ULK1 with ATG13 and RB1CC1 also contributes to its autophosphorylation and protein stability.^51^ Once activated, ULK1 in turn phosphorylates ATG13 (S318/S203), RB1CC1 (S943/S986/S1323), and ATG101 (S11/S203).^52^, ^53^ Further studies are necessary to understand the exact functions of such phosphorylation by ULK1 in autophagy.

Upon activation, the ULK1 complex is recruited to the phagophore assembly site (PAS). So far, how ULK1 complex is recruited remains largely undefined. Both ULK1 and ATG13 contains LC3‐interacting region (LIR), or the ATG8‐interacting motif, which binds to mATG8 (mammalian ATG8, including LC3A/MAP1LC3A (microtubule associated protein 1 light chain 3 alpha), LC3B, LC3C, GABARAP, GABARAPL1 (GABA type A receptor‐associated protein like 1) and GABARAPL2).^54^, ^55^ While mutation of the LIR motif in ATG13 shows marginal effects on autophagy initiation,^56^ mutation of the LIR motif in ULK1 causes a significant defect in autophagy initiation,^54^ indicating that the interaction of ULK1 with mATG8 is important for autophagy induction. While lipidation of mATG8 is normally considered as downstream of ULK1 complex during autophagy, it is believed that GABARAP may act upstream to recruit ULK1 complex to initiate autophagy.

Apart from GABARAP, the small GTPase RAB1A (homolog of Ypt1 in yeast) has also been reported to recruit ULK1 complex to PAS in yeast and mammalian cells.^57^, ^58^ As an effector of RAB1A, C9orf72 (C9orf72–SMCR8 complex subunit) interacts with the ULK1 complex and mediates the translocation of ULK1 complex to PAS via RAB1A.^57^ Moreover, the ER contact proteins VAPA (VAMP‐associated protein A) and VAPB also play important roles in ULK1 complex recruitment via direct interaction with ULK1 and RB1CC1.^59^ It appears that multiple mechanisms are involved in ULK1 recruitment and it would be important to understand how these mechanisms are coordinated with each other to precisely regulate autophagy initiation.

#### 2.1.2 The class III lipid kinase complex I (PI3KC3–C1)

PI3KC3–C1 consists of the lipid kinase PIK3C3, the scaffold and potential protein kinase PIK3R4, the regulatory subunit BECN1, and ATG14, and is responsible for production of PtdIns3P/PI3P, a key lipid metabolite essential for autophagy.^24^ The complex adopts a V shape model, with PIK3C3 and PIK3R4 forming the right arm of the V with catalytic functions, and BECN1 and ATG14 forming the left arm of the V with regulatory functions.^25^ PIK3C3 contains a C2 domain, a helical domain, and a kinase domain. The C2 domain is critical for the binding with PIK3R4 as it has a helical insertion that directly contacts the WD40 domain of PIK3R4.^26^ This WD40 domain of PIK3R4 is also a part of the left arm that serves as a docking site for BECN1 and ATG14.^60^ The central coiled‐coil domain of BECN1 regulates its binding to the coiled‐coil regions of ATG14.^27^ NRBF2 has been discovered as the fifth subunit and its interaction with BECN1 and ATG14 is triggered upon autophagy induction such as amino acid starvation, resulting in enhanced kinase activity and dimerization of PI3KC3–C1.^61^, ^62^, ^63^ Intriguingly, an inhibitory role of NRBF2 in autophagy is also reported.^64^

Similar to ULK1, the subunits of PI3KC3–C1 (including PIK3C3, BECN1, and ATG14) also contain LIR motif with preferential bindings to GABARAP and GABARAPL1 and such bindings help scaffolding PI3KC3–C1 on membranes to promote efficient autophagosome formation.^65^ Upon autophagy induction, these subunits are phosphorylated by ULK1 including PIK3C3 (S249), BECN1 (S15/30/96/279/337), and ATG14 (S29).^52^, ^66^, ^67^ Phosphorylation of PIK3C3 at S249 enhances its bindings to GABARAP, GABARAPL1, and LC3C.^65^ Phosphorylation of Beclin‐1 (S15) and ATG14 (S29) by ULK1 has been shown to enhance the activity of PI3KC3–C1.^66^, ^67^ A more recent study shows that ULK1 also phosphorylates PIK3R4 (S813/S861/S865/S879/S1039/S1289) and mutations of these sites impair the activity of PI3KC3–C1 and autophagy.^68^ Importantly, mutation of the ATG14 LIR motif inhibits the phosphorylation of ATG14 at S29 and BECN1 at S96, thereby inhibiting the activity of PI3KC3–C1.^65^ It appears that the binding of ATG14 to GABARAP and GABARAPL1 is essential for PI3KC3–C1 recruitment and activation following autophagy induction, which is supported by earlier studies which show that ATG14 targets PI3KC3–C1 to PAS.^28^, ^29^ Recruitment and activation of PI3KC3–C1 results in phosphorylation of phosphatidylinositol to produce PtdIns3P essential for autophagosome nucleation and recruitment of downstream effectors.^69^, ^70^

#### 2.1.3 The WIPI family and WIPI–ATG2A complex

Following the activation of PI3KC3–C1 and production of PtdIns3P, ZFYVE1/DFCP1 (zinc finger FYVE‐type containing 1), and the WIPI/PROPPIN (β‐propellers that bind polyphosphoinositides) family act as PtdIns3P effectors to recruit downstream autophagy machinery.^69^, ^71^ Although DFCP1 puncta has been widely used as a marker for omegasome, its functions in autophagy remains largely undefined. On the other hand, WIPI proteins bind to downstream effector proteins via their seven bladed β‐propellers and bind to PtdIns3P via their Phe‐Arg‐Arg‐Gly (FRRG) motifs.^72^, ^73^ There are four members of the WIPI family including WIPI1, WIPI2, WIPI3/WDR45B, and WIPI4/WDR45.^74^ Among these WIPI proteins, the role of WIPI2 in autophagy is relatively well established. Six isoforms of WIPI2 have been reported thus far.^30^, ^75^ Among them, WIPI2B and WIPI2D have been shown to be essential for recruitment of the ATG12–ATG5–ATG16L1 complex to mediate the conjugation of LC3B to PE to promote phagophore expansion (and possibly sealing).^31^, ^75^ Consistently, downregulation or dysfunction of WIPI2 has been linked to defective autophagy.^76^, ^77^ Recently, it has been shown that WIPI2 positively regulates the proteasomal degradation of outer mitochondrial proteins and mitophagy via recruiting VCP (valosin‐containing protein) to damaged mitochondria.^78^ Thus, such observations advance our understanding on WIPI2 functions and reveals the critical role of WIPI2 in the ubiquitin‐proteasome and autophagy–lysosome systems to maintain cellular homeostasis.

As for other WIPIs, WIPI1 was reported to function upstream of LC3B lipidation, while its exact mechanism remains unknown.^79^ WIPI3 and WIPI4 were reported to function upstream of autophagy via regulating STK11/LKB1 (serine/threonine kinase 11)–AMPK (AMP‐activated protein kinase)–TSC2 (TSC complex subunit 2) axis.^32^, ^80^ Recently, it has been reported that WIPI3, similar to WIPI2, is also able to interact with ATG16L1 to promote LC3B lipidation.^75^ Accumulating evidence from recent studies show that both WIPI3 and WIPI4 can interact with ATG2A via the WIR (WIPI‐interacting‐region) motif.^33^, ^81^, ^82^, ^83^ Further studies show that the WIPI3/WIPI4–ATG2A complex serve to establish the contact sites between ER and phagophore, enabling the transportation of lipids to promote phagophore expansion.^33^, ^34^, ^35^ Moreover, WIPI3/WIPI4–ATG2A complex is also involved in autophagosome–lysosome fusion,^84^ adding to the complexity of the WIPIs functions.

Up to date, how WIPI proteins are recruited to omegasome, the phagophore nucleating from a subdomain of the ER, is less understood. RAB11A (RAB11A, member RAS oncogene family) has been reported to recruit WIPI2 to PtdIns3P‐enriched membrane structures to mediate LC3B lipidation.^85^ TRAPPC11 (trafficking protein particle complex subunit 11) has been shown to recruit WIPI4–ATG2A complex to promote phagophore expansion.^86^ Whether WIPI3–ATG2A complex is recruited via a similar mechanism remains to be further tested.

#### 2.1.4 The two ubiquitin‐like conjugation systems

The two ubiquitin‐like conjugation systems refer to the mATG8 and ATG12 systems in which mATG8 is conjugated to PE, while ATG12 is conjugated to ATG5 in a ubiquitination‐like manner.^38^, ^39^ mATG8 is firstly cleaved by ATG4 and thereby exposes a glycine residue.^87^, ^88^ Among the four isoforms of ATG4 reported, ATG4B is able to cleave all members of the mATG8, while ATG4A is more specific for GABARAPs.^36^, ^37^ Cleaved mATG8 is subsequently conjugated to PE with the action of ATG7 (E1), ATG3 (E2) and the ATG12–ATG5–ATG16L1 complex (E3).^4^ In the ATG12 system, the covalent conjugation of ATG12 to ATG5 relies on ATG7 (E1) and ATG10 (E2), and the resultant ATG12–ATG5 conjugates further interact with ATG16L1 to form the ATG12–ATG5–ATG16L1 complex that acts as a E3‐like enzyme to conjugate mATG8 to PE.^40^ mATG8 lipidation is essential for phagophore expansion and possibly closure.^89^, ^90^

While the recruitment of ATG16L1 has been reported to be mediated by WIPI2, it has been recently showed that ATG16L1 contains two membrane‐binding regions and can support mATG8 lipidation even in the absence of WIPI2.^40^ In addition, ULK1‐mediated phosphorylation of ATG16L1 at S278 is important for this recruitment. However, whether this phosphorylation is important for ATG16L1 interaction with WIPI2 needs to be further investigated.

#### 2.1.5 ATG9 vesicles

ATG9 is the only transmembrane protein of the autophagy core machineries and is predicted to contain six transmembrane helices.^43^ There are two members of ATG9 in mammalian system including ATG9A and ATG9B, which are the main connection between the Golgi complex and autophagosome biogenesis.^91^ ATG9 resides in the Golgi complex and follows the traffic via the endosomal system including early, sorting, and late endosomes.^44^, ^45^ As a result, the ATG9 vesicles are believed to be derived from the Golgi complex and the Golgi–endosomal system.^44^, ^45^ The ATG9 vesicles are mobilized to PAS by TRAPPIII (trafficking protein particle III) complex.^92^ Phosphorylation of ATG9 at S761 is mediated differentially by ULK1 and AMPK under basal and stress conditions and is required for ATG9 localization at autophagosomal structures.^93^

At present, the exact functions of the ATG9 vesicles have been one of the leading questions in the autophagy field and remain mysterious. The ATG9 vesicles are believed to contribute to autophagosome expansion via supplying the membrane source.^44^, ^94^ Such a notion is supported by the observations in yeast that ATG9 vesicles function as seeds for phagophore growth.^95^ Further studies are needed to further understand the precise functions of ATG9 in autophagosome biogenesis.

### 2.2 Autophagosome–lysosome fusion

The final step of autophagy is the fusion between the double‐membrane autophagosome and lysosome, which leads to degradation of the autophagic cargo by the lysosomal hydrolases (Figure 1).^96^ This process is regulated by a series of factors including: (i) phosphoinositides, such as PtdIns3P; (ii) small GTPases, such as RAB7; (iii) mATG8 proteins; (iv) tethering factors, such as the HOPS (homotypic fusion and vacuole protein sorting) tethering complex; (v) SNARE (SNAP (Soluble NSF Attachment Protein) receptor) complexes; (vi) motor adaptors, such as PLEKHM1 (pleckstrin homology and RUN domain containing M1).^97^ Due to space restraint, we will focus the discussion on the tethering complex and the SNARE complex.

#### 2.2.1 The HOPS complex

The HOPS complex is the core tethering complex for autophagosome–lysosome fusion. It consists of six subunits, including VPS11, VPS16, VPS18, VPS33A, VPS39, and VPS41.^98^ All these subunits are able to interact with STX17 (syntaxin‐17), one of the key SNARE proteins present at the completed autophagosome.^99^ On the other hand, RABs are small GTPases to mediate membrane fusion via binding to tethering factors.^100^ Among them, RAB7 is the best studied for autophagosome–lysosome fusion. RAB7 is localized to late endosomes and lysosomes.^101^, ^102^ Importantly, RAB7 binds to two subunits of HOPS, VPS39, and VPS41.^103^, ^104^ Formation of the RAB7–HOPS complex is critical for membrane tethering to support SNAREs‐mediated autophagosome–lysosome fusion.^105^

The molecular mechanisms underlying the recruitment of HOPS complex to autophagosome remain largely unknown. UVRAG (UV radiation resistance associated) is a regulatory subunit of PI3KC3‐C2 (the class III lipid kinase complex II) which is involved in autophagosome–lysosome fusion.^25^ This complex has been shown to interact with and recruit the HOPS complex to autophagosome to promote autophagosome–lysosome fusion.^106^ Moreover, a study from Dikic's laboratory reveals a RAB7 effector protein PLEKHM1 as a key positive regulator for the HOPS complex recruitment via simultaneous binding to LC3 and RAB7.^104^ The exact function of the HOPS tethering complex in the process of autophagosome–lysosome fusion remains to be further investigated, especially in model organisms such as yeast and drosophila.

#### 2.2.2 SNAREs

SNAREs are the main players in control of membrane‐mediated transport via vesicle fusion^107^ and have been shown to be the core machinery for autophagosome–lysosome fusion.^108^ Structurally, SNAREs can be classified into Q‐SNAREs (those having a Gln/Q residue) and R‐SNAREs (those having an Arg/R residue).^109^ The Q‐SNAREs can be furthered classified into Q‐a, Q‐b, and Q‐c SNAREs based on the amino acid sequence within the SNARE domain.^110^, ^111^ The R‐SNARE and Q‐SNARE protein on separate membranes form the trans‐SNARE complex for membrane fusion.^108^ For instance, the Qa‐SNARE protein STX17 localizes to the autophagosome, the R‐SNARE protein VAMP8 (vesicle‐associated membrane protein 8) localizes to lysosome/late endosome and by recruiting the Qbc‐SNARE protein SNAP29 (synaptosome associated protein 29), these SNAREs form the trans‐SNARE complex to mediate autophagosome–lysosome fusion.^108^ Other trans‐SNARE complexes, including STX17–SNAP29–VAMP7 and YKT6 (YKT6 v‐SNARE homolog)–SNAP29–STX17, are also reported to regulate autophagosome–lysosome fusion.^97^

In addition to HOPS and SNAREs, one key ATG, ATG14, has also been shown to play a key role in regulating autophagosome–lysosome fusion: ATG14 binds to STX17, which contributes to stabilization of STX17–SNAP29 complex and promotes membrane tethering and enhancing membrane fusion.^112^ Given that phosphorylation of STX17 regulates its functions^113^ and that ATG14 is a key component of the ULK1 complex,^50^ it would be interesting to test whether ULK1 plays a role in control of STX17 phosphorylation and functions. O‐linked beta‐N‐acetylglucosamine (O‐GlcNAc) modification of SNAP29 has been reported to undermine its association with STX17, which inhibits the formation of the trans‐SNARE complex.^114^ Of note, O‐GlcNAcylation of SNAP29 is controlled by nutrient status and is reduced by starvation.^114^ More studies are needed to better understand how precisely SNAREs are recruited and how their functions are regulated in the course of autophagosome–lysosome fusion.

### 2.3 Upstream regulators of autophagy

MTORC1 (mammalian target of rapamycin complex 1) and AMPK are the two key regulatory factors that work upstream in control of autophagy. In the following sections, we will focus on these two key protein kinases and their roles in regulation of autophagy.

#### 2.3.1 MTORC1

MTOR is a serine/threonine protein kinase belonging to PI3K‐related protein kinases family that responds to growth factor and nutrients.^115^ In mammalian cells, MTOR forms two distinctive complexes known as MTORC1 and MTORC2, with MTOR as the catalytic subunit.^116^ MTORC1 is assembled by MTOR, MLST8 (MTOR‐associated protein, LST8 homolog), RAPTOR (regulatory associated protein of MTOR complex 1), DEPTOR (DEP domain‐containing MTOR‐interacting protein), PRAS40 (proline‐rich Akt substrate 40), and FKBP12/FKBP1A (FKBP prolyl isomerase 1A), while MTORC2 is assembled by MTOR, MLST8, DEPTOR, RICTOR (RPTOR independent companion of MTOR complex 2), MAPKAP1 (MAPK associated protein 1), and PRR5/5L (proline rich 5/5L).^46^, ^116^

The role of MTORC1 in autophagy has been extensively studied over the past decades and it has been revealed as a crucial negative regulator for autophagy. MTORC1 can be activated via various stimuli. For example, in response to amino acid, MTORC1 is activated by the GTP‐bound RHEB (ras homolog enriched in brain) and lysosomal translocation via a protein complex called regulator and V‐ATPases.^115^ In response to growth factor such as insulin, PIK3CA–AKT pathway is activated, which then inhibits TSC1/2 (tuberous sclerosis 1/2) and release RHEB (Ras homolog enriched in brain), a small GTPase, from this complex. Subsequently, the active GTP‐bound RHEB mediates the activation of MTORC1.^117^ MTORC1 suppresses autophagy mainly through the following mechanisms: (i) phosphorylation of the autophagosome core machineries including ULK1 (S637/S757), ATG13 (S258), ATG14 (S3/S383/S440/T233), NRBF2 (S113/S120), and WIPI2 (S395) to inhibit autophagosome biogenesis^118^; (ii) phosphorylation of TFEB (transcription factor EB) (S122/S142/S211) to transcriptionally suppress autophagy.^119^ As a result, various MTOR inhibitors (such as rapamycin) have been reported to effectively induce autophagy.^120^

#### 2.3.2 AMPK

AMPK is a serine/threonine kinase and exists as an obligate heterotrimer consist of a catalytic subunit (α) and two regulatory subunits (β and γ).^121^ AMPK is an important cellular energy sensor that can be activated by phosphorylation at T172 mediated by STK11/LKB1 (serine/threonine kinase 11) upon glucose deprivation.^122^ AMPK positively regulates autophagy via multiple mechanisms. First, AMPK inhibits MTORC1 directly via phosphorylating RAPTOR (S722).^123^ Second, AMPK activating TSC2 via phosphorylation (T1227/S1345), thereby inhibiting MTORC1 indirectly.^124^ Third, AMPK promotes autophagy by activating the ULK1 complex or PI3KC3‐C1 via phosphorylating ULK1 (S317/S777),^125^ BECN1 (S91/S94/T388),^126^, ^127^ and ATG13 (S224).^128^

Interestingly, AMPK‐mediated phosphorylation of other targets could also inhibit autophagy. For instance, phosphorylation of PIK3C3 (T163/S165) by AMPK suppresses the production of PtdIns3P, which could be reversed by overexpression of ATG14.^127^ Moreover, it has been reported that WIPI4–ATG2A complex interacts with AMPK under fed condition, while upon glucose deprivation, WIPI4–ATG2A complex disassociates from AMPK and is recruited to nascent autophagosome.^32^ While the biological implication of such interaction has not been studied, findings from this study raised the possibility that AMPK may inhibit the WIPI4–ATG2 complex function via phosphorylation under fed condition.

## 3 IMPLICATION OF AUTOPHAGY IN HEALTH AND DISEASES

As one of the most intricate processes in cells, the roles of autophagy has been very fascinating and is not limited to a basic starving response, but also extended to various “controversial pair” roles like “antiapoptotic versus proapoptotic,”^129^ “antibacterial versus probacterial,”^130^, ^131^ and “antitumorigenic versus protumorigenic.”^132^, ^133^ As a result, dysregulation of autophagy is closely associated with various human diseases.^16^ In this section, we will discuss the recent insights into the role of autophagy in the most pervasive human diseases, including cancer, neurodegenerative diseases, metabolic diseases, viral infections, cardiovascular diseases, and aging. Understanding the implication of autophagy in these diseases is crucial for moving autophagy research from bench to bed for clinical applications in diagnosis and therapy.

### 3.1 Autophagy in cancer

Autophagy has long been considered as a double‐edged sword in cancer. At the early initiation stage, autophagy acts as tumor suppressor via removal of potentially harmful cytosolic contents and damaged organelles, thereby avoiding cell injury such as DNA mutation. At the stage of progression, autophagy acts as a survival mechanism to sustain tumor viability under stressful microenvironment, which also contributes to therapeutic resistance. Therefore, in‐depth understanding of the roles of autophagy during various stages of carcinogenesis and in tumor therapeutic responses will provide important therapeutic strategies to eliminate cancer cells, reverse drug resistance and prevent recurrence.

#### 3.1.1 Autophagy in cancer initiation and progression

The discovery of a monoallelic deficiency of the key autophagy gene *BECN1* in breast, ovarian, and prostate malignancies led to the hypothesis that autophagy is a tumor suppressive mechanism.^134^, ^135^ Such hypothesis is supported by studies in cell‐based assays and animal models which showed enhanced tumorigenesis via genetical suppression of autophagy.^136^, ^137^ Consistently, pharmacological interventions to block autophagic flux also results in the appearance of early tumor lesions in various preclinical tumor models.^138^ The anticancer effects have been linked to the roles of autophagy in various cellular processes, including maintaining genomic integrity, suppressing oxidative stress, and inhibiting NRF2/NFE2L2 (nuclear factor (erythroid‐derived 2)‐like 2) overactivation.^16^ For example, Holdgaard et al.^139^ identified selective autophagy as an essential centrosome‐regulating process in mitosis. Moreover, increasing evidence supports the notion that the oncosuppressive functions of autophagy are partly due to its ability to attenuate the inflammatory response and counteract the establishment of an inflammatory milieu.^140^, ^141^, ^142^, ^143^

Different from the above studies, there are reports showing that autophagy promotes initiation, progression or adaptive responses in tumor cells, indicating the protumor function of autophagy. For instance, DeVorkin et al.^144^ found that tumor development was greatly slowed in mice deficient in Atg5, Atg14, or Atg16L1. Yamamoto et al.^145^ discovered that inhibiting autophagy enhances antitumor T cell responses and therefore suppresses tumor growth. Vera‐Ramirez et al.^146^ reported that autophagy is a crucial strategy for the survival of disseminated dormant breast cancer (BC) cells, while inhibition of autophagy in quiescent BC cells causes apoptosis. These conflicting functions may reflect a fact that the exact role of autophagy in cancer depends on an array of factors, including the stage of the tumor, the type of the tumor and genetic context of the host such as the mutated status of *p53*.^147^

#### 3.1.2 Autophagy in tumor immunosurveillance

**FIGURE 2:**
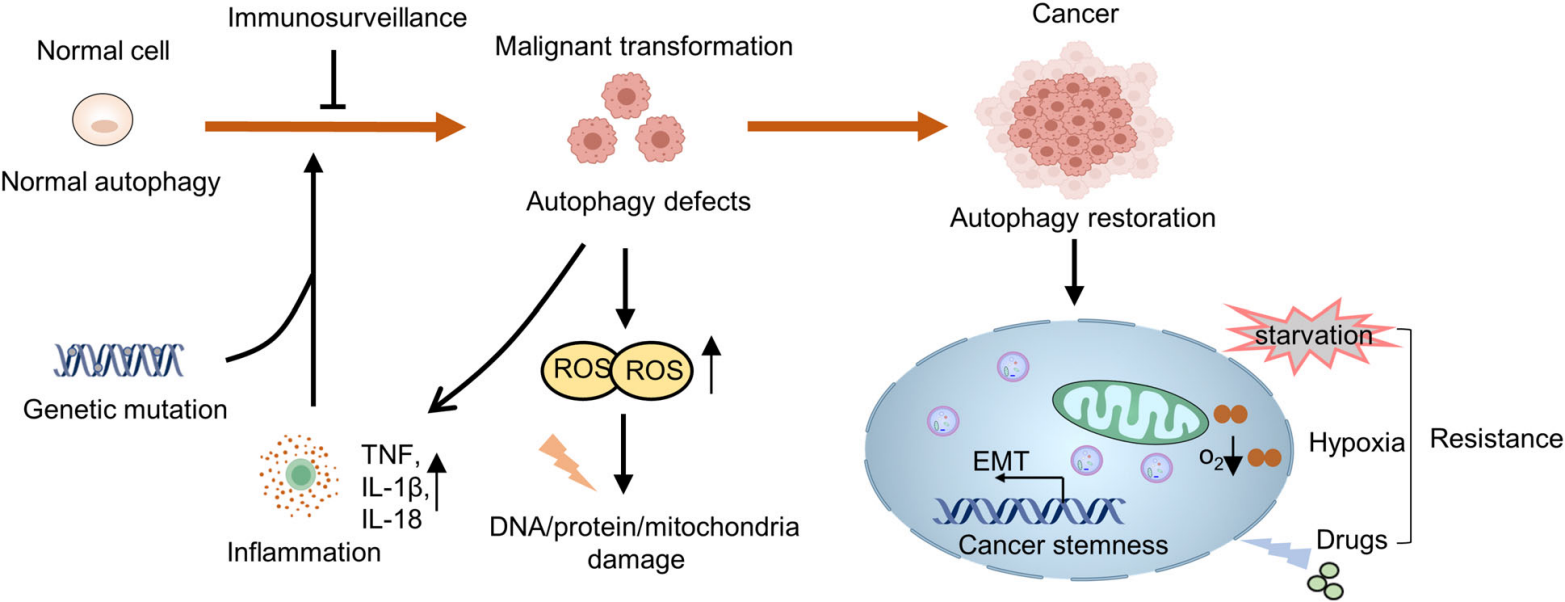
The implication of autophagy in carcinogenesis. Autophagy may act as a powerful barrier to prevent transformation of normal cells into tumor cells. Upon autophagy deficiency, misfold proteins or dysfunctional organelles accumulates, which may cause genomic defects that accompanies transformation. On the other hand, autophagy acts as a mechanism for transformed cells to get adapted to various cellular stress responses, promote metastasis, and maintain tumor stemness

The roles of autophagy in preventing malignant transformation have been linked to its functions in regulation of the microenvironment and activation of immunosurveillance (Figure 2).

As autophagy is essential for the removal of cytotoxic contents to maintain the intracellular homeostasis, impaired autophagy may result in accumulation of cytotoxic factors and the subsequent formation of premalignant microenvironment.^148^ One good example to support such notion is the generation of reactive oxygen species (ROS) due to impaired autophagy.^149^ ROS causes cell injury via multiple mechanisms such as modification and dysfunctions of proteins, DNA or lipids, and increased risk of DNA mutation, all of which increase the risk of malignant transformation.^150^ Additionally, ROS is also involved in production of proinflammatory cytokines such as tumor necrosis factor.^140^ As autophagy is a main pathway for inflammasomes degradation, impairment of autophagy further exacerbates inflammation via promoting maturation and secretion of proinflammatory factors such as IL‐Iβ and IL‐18.^151^ Moreover, excessive ROS also impairs mitochondria and promotes the release of mitochondrial DNA, which also induces the expression of inflammation‐related genes via activating the cGAS (cyclic GMP–AMP synthase)–STING (stimulator of interferon response cGAMP interactor 1) signaling pathway.^152^, ^153^ Chronic inflammation has been found as a key factor that contributes to malignant transformation. These findings together suggest the importance of autophagy in fine‐tuning inflammation and prevents the formation of pre‐malignant microenvironment.

Elimination of malignant cells by immune cells is another key part of immunosurveillance. Autophagy indeed plays multiple roles in regulating immune cell functions. First, autophagy is actually involved in renewal, differentiation, and homeostasis of immune cells, including dendritic cells (DCs) and T cells.^154^, ^155^ Second, autophagy also modulates MHC class I and class II antigen presentation to promote cancer cell death.^156^ Third, autophagy is also essential for long‐term survival of CD8+ cytotoxic T lymphocytes upon acquisition of a memory phenotype.^157^ Consistently, defective autophagy is associated with reduced immune response.^158^, ^159^ The roles of autophagy in immunosurveillance may thus contribute to prevention of malignant transformation.

#### 3.1.3 Autophagy in cancer stem cells

Cancer stem cells (CSCs) refer to a group of cancer cells that possess characteristics of stem cells with an ability to form a new cancer. CSCs are usually featured with high potency in proliferation, resistance to therapeutics, and are responsible for metastasis and relapse of cancer.^160^ Determining the molecular mechanisms underlying CSC survival is therefore critical for overcoming the current challenges in anticancer therapies. Although the concept of CSCs is still in dispute, over the recent decade, the functional implication of autophagy has been suggested in CSCs from a wide variety of tissues, including pancreas, liver,^161^ breast,^162^ ovarian,^163^ and brain.^164^, ^165^ In most cases, it is believed that autophagy defects impair the self‐renewal capacity of CSCs.^166^ Furthermore, autophagic flux has been revealed to be significantly higher in CSCs than in bulk tumor cells.^162^, ^167^ Consistently, increased autophagic flux has been linked to CSCs‐mediated tumorigenesis, such as the development of leukemia^168^ and BC.^162^ Furthermore, autophagy can regulate the stemness of tumor cells,^169^ and suppression of autophagy with chloroquine (CQ) reduces the CSCs populations and thereby sensitizes cancer cells to chemotherapy.^170^ Intriguingly, another study shows that autophagy suppresses the CSCs properties of glioma cells.^171^ As autophagy and tumor stemness maintenance are closely related to metastasis and chemoresistance, targeting autophagy and CSCs may be the key direction to prevent tumor resistance and recurrence.

### 3.2 Autophagy in neurodegenerative diseases

Autophagy is essential for maintaining the homeostatic demands of neurons, both at the level of the central and peripheral nervous systems.^172^, ^173^ Based on the observations that neurodegenerative disorders occur in autophagy‐defective mice, it has been hypothesized that autophagy defect is an important etiological factor for neurodegenerative diseases in humans. Pathologically, most neurodegenerative diseases are associated with accumulation of protein aggregates, including mutant α‐synuclein in Parkinson disease (PD), Aβ and C‐terminal fragments of the amyloid precursor protein (APP) in Alzheimer disease (AD), pathogenic mutant huntingtin (mHtt) in Huntington disease (HD), and mutant SOD1 (superoxide dismutase 1) as well as TDP‐43/TARDBP (TAR DNA binding protein) in amyotrophic lateral sclerosis (ALS).^172^, ^174^ These protein aggregates are toxic drivers of neurological lesions and are supposed to be degraded by the autophagy–lysosome pathway.^172^ Consistently, gene mutations of autophagic receptors (e.g., SQSTM1 (sequestosome 1), OPTN (optineurin), NBR1 (neighbor of BRCA1 gene 1)) are closely associated with neurodegenerative diseases.^175^, ^176^, ^177^ As a result, modulating autophagy is believed to be a promising strategy for treating neurodegenerative diseases. For example, autophagy activation of the aggregating receptor SQSTM1 promotes the clearance of mHtt, insoluble tau, and Aβ42.^178^, ^179^ Conversely, inhibition of autophagy with 3‐methyladenine (3‐MA) results in accumulation of mHtt aggregates.^180^, ^181^ Here, we review the recent advances in understanding the pathogenesis of neurodegenerative diseases associated with defective autophagy and discuss the therapeutic interventions for these diseases via targeting autophagy.

#### 3.2.1 Alzheimer disease

AD, the most common neurodegenerative disorder, is characterized by the progression from episodic memory problems to severe cognitive decline.^182^, ^183^ So far, no effective therapy is available to block or slow down AD progression, and the exact mechanisms underlying the disease remain mysterious.^184^ Intracellular microtubule associated protein tau (MAPT)/tau tangles and extracellular beta amyloid peptide [Ab] plaques in the brains of patients with AD are the early pathological features that gradually lead to neuronal cell death and cognitive decline.^17^ In fact, the connections between autophagy and AD originated from the observation of accumulated immature autophagic vacuoles in AD brains.^185^ Multilayered brain proteomic analysis revealed that the autophagy cargo receptor SQSTM1 accumulates in AD, suggesting impaired autophagic flux.^186^ Moreover, functional autophagy is required for removal of soluble and aggregated variants of MAPT/tau.^187^, ^188^ Consistently, genetic variations of CTSD (cathepsin D), a lysosomal peptidase involved in clearance of aggregated proteins such as tau, is associated with an increased risk of AD.^189^ Clearly, these studies indicate the important role of autophagy in AD via targeting MAPT/tau for lysosomal degradation. Interestingly, accumulating MAPT/tau tangles can in turn perturb the retrograde axonal transport of autophagosomes by interfering with the dynein–DCTN (dynactin) complex, ultimately triggering the deleterious accumulation of MAPT/tau‐containing autophagic vesicles.^190^

Aβ originates from the processing of its APP and its accumulation is also a hallmark of AD.^191^ Autophagy is known to play an important role in Aβ quality control. First, it has been found that induction of ATG5‐dependent autophagy enhances the degradation of APP.^192^ Second, autophagy also modulates Aβ clearance. Enhancing autophagy by chemical reagents or genetic engineering techniques significantly reduces the deposition of intracellular Aβ and extracellular amyloid in mice brain and improves cognitive ability.^193^, ^194^, ^195^ Moreover, activation of autophagy reduces the burden of Aβ plaque in rodents.^172^, ^196^, ^197^ Third, it has been revealed that autophagy is not only required for intracellular degradation of Aβ via lysosome, but also for Aβ secretion.^198^, ^199^ These results thus suggest that autophagy plays multiple roles in intracellular Aβ clearance.

In the course of AD pathogenesis, accumulated Aβ leads to ROS production which causes mitochondrial damage, and mitophagy is the essential mechanism to eliminate damaged mitochondria.^14^, ^183^, ^200^ In fact, mitochondrial dysfunction is also defined as one of the early pathological features of AD. This is supported by the evidence from AD postmortem brain tissues that mitochondrial dysfunction is one of the initial pathological events in AD.^201^ Similarly, mitophagy is impaired in induced pluripotent stem cell‐derived human AD neurons, animal AD models, and the hippocampus of AD patients.^183^, ^202^ As a result, mitophagy activation alleviates both Aβ and tau pathologies and improves the cognitive functions of *Caenorhabditis elegans* and mouse AD models.^203^ Although further investigation is required to elucidate the relationship between mitophagy and AD pathogenesis, mitophagy‐mediated clearance of dysfunctional mitochondria appears to be a promising therapeutic target for AD intervention.

#### 3.2.2 Parkinson disease

**FIGURE 3:**
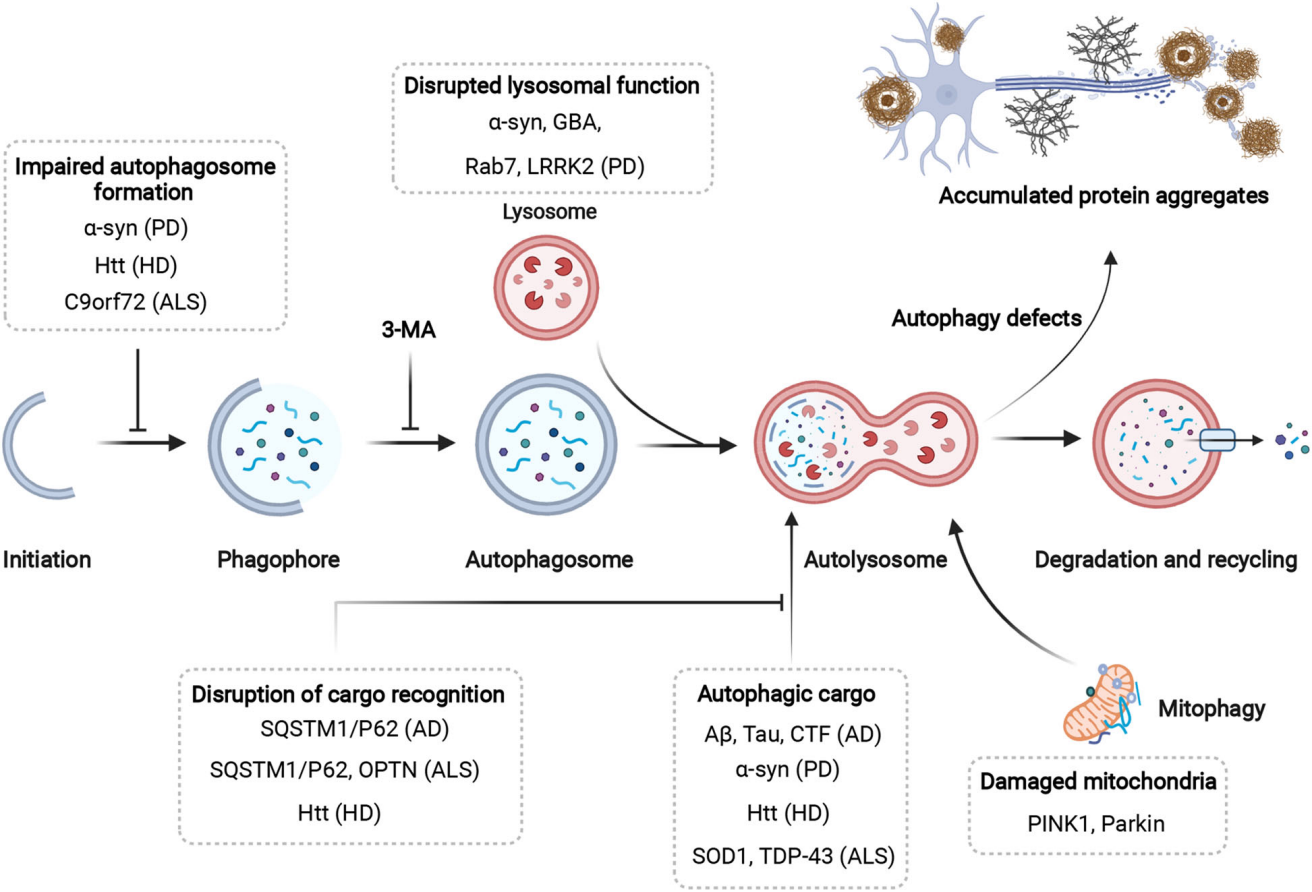
The mechanisms and therapeutic targets of autophagy in neurodegenerative diseases. An increasing number of genes associated with neurodegenerative diseases, especially AD, PD, HD, and ALS, act at different steps throughout the autophagic process. Their proposed sites of action, as well as associated neurodegenerative diseases are indicated. The protein aggregates, supposed to be degraded by the autophagy–lysosome pathway, accumulate and contribute to neurodegenerative diseases when autophagy is defective. AD, Alzheimer's disease; PD, Parkinson's disease; HD, Huntington's disease; ALS, Amyotrophic lateral sclerosis

PD is the second most common neurodegenerative disease.^204^ PD is pathologically defined by the loss of dopaminergic neurons in the substantia nigra (SN) and the prevalence of proteinaceous Lewy bodies, characterized by pathological α‐synuclein inclusions in the dopaminergic neurons of the SN.^17^, ^205^ It is well known that α‐synuclein carrying pathogenic mutations are degraded by the autophagy‐lysosome system.^206^ Knockout of *Atg7* induces an age‐dependent increase in the formation of Sqstm1 α‐synuclein inclusion bodies in dopaminergic neurons of aged mice, leading to motor dysfunction.^207^ Conversely, α‐synuclein inclusions impair the autophagic pathway.^208^, ^209^, ^210^, ^211^ These studies collectively suggest the close correlation between defective autophagy and α‐synuclein aggregates accumulation (Figure 3).

Glucocerebrosidase (GBA) is a lysosomal enzyme for degrading glucosylceramide, and mutations in *GBA* are one of the most common genetic risk factors for PD.^212^ PD‐associated GBA mutations (N370S and L444P) reduced its protein levels and enzymatic activity and impaired its trafficking from the ER to the lysosomes, leading to ER stress, accumulation of lysosomal lipids, and ultimately autophagy–lysosome dysfunction.^213^, ^214^ It has been demonstrated that the GBA interacts with α‐synuclein to promote α‐synuclein degradation and prevent α‐synuclein aggregation, while lysosomal membrane‐bound α‐synuclein is able to inhibit lysosomal functions and further exacerbates PD.^215^, ^216^

Besides, mutations in LRRK2/PARK8 (leucine‐rich repeat kinase 2) are genetically linked to autosomal‐dominant PD and have also been extensively studied for its involvement in aberrant autophagy. More than 40 pathogenic LRRK2 mutations have been reported in patients with PD.^217^ Loss of LRRK2 function leads to striking defects in the endolysosomal and autophagy pathways.^218^ Interestingly, many pathogenic mutations in LRRK2 are gain‐of‐function mutations, such as G2019S and R1441C, which increase its kinase activity but impair autophagic degradation, similar to LRRK2 deficiency.^219^, ^220^, ^221^ Further studies are needed to understand the exact roles of LRRK2 in autophagy and its implication in PD pathogenesis.

Mutations in LRRK2, α‐synuclein, and GBA contribute to increased or impaired autophagy, but they also contribute to mitochondrial dysfunction.^204^ It had been shown that patient cells with G2019S mutation in LRRK2 showed an increase in the number of mitochondrial fragments and a decrease in mitochondrial membrane potential.^222^, ^223^ Ryan et al.^224^ demonstrated that neurons expressing mutant α‐synuclein also exhibit fragmented mitochondria, as well as exposed cardiolipin on outer mitochondrial membrane, which initiates mitophagy. Patients with GBA1 deficiency and mouse models of *GBA1* knockout exhibit mitochondrial dysfunction.^225^, ^226^, ^227^

Given the close association of mitochondrial dysfunction with PD, defective mitophagy (selective autophagy of damaged mitochondria) has emerged as one of the main causes contributing to PD progression.^228^ Mutations in the genes encoding for PINK1 (PTEN (phosphatase and tensin homolog)‐induced putative kinase‐1) and Parkin have been found in some familial forms of PD.^229^ The field of PINK1–Parkin‐dependent mitophagy has significantly expanded over this decade. In this pathway, PINK1 is stabilized and accumulates on the mitochondria upon mitochondria depolarization. Subsequently, ubiquitin phosphorylated by PINK1 at Ser65 serves as docking sites for Parkin binding, which partly activate Parkin E3 ligase activity.^230^, ^231^, ^232^ Subsequent phosphorylation of Parkin by PINK1 within its ubiquitin‐like domain fully activates its E3 ligase activity.^233^, ^234^ In this way, PINK1 and Parkin form a feedforward loop to amplify the mitophagy signal to mediate clearance of damaged mitochondria.^235^, ^236^ Impairment of PINK1–Parkin signaling pathway leads to accumulation of damaged mitochondria, which causes oxidative stress and toxic burdens that could lead to neuronal cell death.^228^ A recent study from Youle's group^237^ reports a strong inflammatory phenotype in both the PINK1−/− and Parkin−/− mice due to the release of mtDNA and the subsequent activation of the cGAS–STING pathway. While the exact roles of PINK1 and Parkin in the pathogenesis of PD is still not well understood given the fact that mice lacking either PINK1 or parkin do not develop substantial PD‐relevant phenotypes, this study implies that PINK1–Parkin‐dependent mitophagy mitigates PD via fine‐tuning innate immunity response.

#### 3.2.3 Huntington disease

HD is an incurable, autosomal‐dominant progressive neurodegenerative disease, which is mainly manifested by cognitive dysfunction, behavioral disturbances, and severe motor dysfunction.^238^ HD is caused by the expansion of the CAG repeat within a single gene *huntingtin (HTT)*, which encodes a large protein with an extended polyglutamine (polyQ) tail.^239^ The abnormal expansion of a polyQ repeat in exon 1 produces mHtt proteins, leading to cytotoxicity in the striatum and cortex and inducing progressive motor deficits accompanied by the accumulation of autophagosomes.^240^, ^241^ There is substantial evidence that autophagy is dysfunctional in HD. For example, it has been found that expression of the truncated N‐terminal huntingtin fragment (HTT552) activates the autophagy/lysosomal degradation pathway via increasing LC3‐II, BECN1, and CTSB/CTSL expression.^242^ Importantly, significant differences have emerged between the functions of wild‐type and mutant HTT in regulating the autophagy process.^243^ Compared with wild‐type HTT, mutant HTT has multiple roles in autophagy inhibition.^244^ For instance, Martinez‐Vicente et al.^245^ found that mutant HTT negatively affects autophagosomal cargo recognition through dysregulated recruitment of SQSTM1. Rui et al.^246^ demonstrated that mutant HTT disrupts the ability of HTT to bind and activate ULK1.

In fact, mHtt aggregation status and HD progression can be modulated by autophagy.^179^, ^247^ Through a mouse model, it has been reported that heterozygous loss of the autophagy adaptor protein Alfy/Wdfy3 (WD repeat and FYVE domain containing 3) impedes the clearance of mHtt and promotes HD progression.^248^ In an unbiased in vivo genome‐wide screening, Wertz et al.^249^ found that many ATGs appear to prevent mHtt toxicity and HD progression. Taken together, identifying the precise regulatory mechanisms of autophagy in HD will be essential for the development of rational therapeutic interventions via targeting autophagy.

#### 3.2.4 Amyotrophic lateral sclerosis

ALS is a rare and devastating neurodegenerative disease, characterized by the selective death of motor neurons controlling the voluntary muscles.^250^, ^251^ Pathologically, ALS is featured by cytoplasmic ubiquitin‐positive inclusion formation in the brain and spinal cord and the aberrant amassing of misfolded proteins including SOD1 and TDP‐43.^17^ Besides, inherited genetic mutations in TDP‐43, sarcoma (FUS), and UBQLN2 (ubiquilin 2) also cause ALS.^252^, ^253^ Numerous studies suggest that autophagy is closely implicated in the pathogenesis of ALS. For example, autophagy induction mitigates ALS via TDP‐43 clearance.^254^ Aggregation‐prone SOD1 and TDP‐43 fail to be disposed of upon mutation of SQSTM1.^175^, ^255^ Moreover, SQSTM1 was also found to colocalize with TDP‐43 inclusions and its deficiency exacerbated ALS symptoms caused by the insoluble protein aggregates.^256^, ^257^ UBQLN2, a genetic risk factor for ALS, is also closely associated with autophagy. It has been reported that UBQLN2 forms a complex with LC3 and facilitates autophagosome–lysosome fusion.^258^ Besides, Chen et al.^259^ demonstrated that selectively expressing mutant UBQLN2^P497H^ in motor neurons compromises autophagy–lysosome fusion and is sufficient to trigger ALS progression in rats. Conversely, interventions to activate autophagy, such as depletion of the transcription factor XBP1 (X‐box binding protein 1) and pharmacological modulation of HSPB8 (heat shock protein family B (small) member 8) expression in the nervous system, counteract ALS symptomatology by promoting autophagic clearance of SOD1.^260^, ^261^ These studies clearly suggest the importance of autophagy in the clearance of misfolded proteins that contribute to ALS progression.

In addition, C9orf72 (hexonucleotide repeat amplification of the intron of Chromosome 9 ORF 72) is the most common genetic cause for ALS.^262^ Compared with healthy individuals, ALS patients typically carry more than 100 hexanucleotide repeats.^263^ Although the cellular function of C9orf72 remains unclear, increasing evidence has suggested that C9orf72 affects ALS pathogenesis by modulating autophagy via various mechanisms. It has been reported that C9orf72 functionally interacts with multiple members of the Rab small GTPases family to positively regulate autophagy, while loss of C9orf72 impairs autophagosome biogenesis in neuronal cells.^264^ C9orf72 is also reported to be associated with the autophagy core machinery such as the RB1CC1–ULK1 complex to promote autophagosome biogenesis.^265^ As a result, deficiency of C9orf72 also leads to autophagic degradation blockade, leading to accumulation of cytotoxic contents and ultimately neuronal cell death.^266^, ^267^ These results suggest an important role of C9orf72 in control of autophagy and its implications in ALS pathogenesis.

### 3.3 Autophagy in metabolic diseases

Overnutrition and reduced energy expenditure, mirrored by aberrant activation of the trophic axis (e.g., insulin signaling), contribute to the development of metabolic diseases such as obesity, insulin resistance, and type 2 diabetes (T2D). Mechanistically, excess fat accumulation causes insulin resistance and elevated serum free fatty acid levels, leading to systemic lipotoxicity and β‐cell dysfunction.^268^ In fact, autophagy responds to minimal oscillations in intracellular and extracellular metabolism, thereby maintaining a tightly regulated balance between the anabolic and catabolic pathways.^269^ For instance, the essential molecular players of cellular energy status, such as MTORC1 and AMPK, are involved in nutrient deprivation‐induced autophagy. Autophagy performs inherent metabolic tasks in major organs such as adipose tissue, liver, and the exocrine pancreas and participates in maintaining energy balance in the body.^270^ As a result, dysregulated autophagic flux contributes to the pathogenesis and progression of metabolic diseases. In this section, due to space limitation, we will focus on the implications of autophagy in obesity and T2D.

#### 3.3.1 Obesity

As a metabolic disorder, obesity is clinically manifested by an excessively sustained positive energy balance. An overly simplistic view holds that the pathogenesis of obesity underlies the dramatic accumulation of autophagic substrates such as lipid droplets, protein aggregates, and damaged mitochondria, and that a defective autophagic process accelerates obesity development.^271^ In fact, this view is easily confuted because multiple intracellular and extracellular factors are closely related to the pathogenesis and progression of obesity.

At present, there is evidence indicating that autophagy is repressed under obesogenic conditions. For instance, in mice with prolonged feeding of a high‐fat diet, the expression levels of Atg5 and Atg7 in the liver are significantly downregulated, indicating that autophagy is impaired.^272^ Consistent with this finding, an obesity‐induced increase in cytoplasmic calcium concentration in hepatocytes impedes fusion between autophagosomes and lysosomes.^273^ Conversely, restoring the expression levels of Atg7 and Tfeb in liver prevents weight gain and metabolic syndrome in diet‐induced and genetically obese mouse models.^272^, ^274^ Furthermore, autophagy can also contribute to appetite. It has been revealed that hypothalamic inhibition of autophagy increased energy intake and reduced energy expenditure.^275^ However, genetic obliteration of *Atg7* in hypothalamus contributes to a lean phenotype.^276^ Intriguingly, autophagy is involved in the conversion of white adipose tissue into brown adipose tissue. Upon adipose‐specific deletion of *Atg7*, the white adipose tissue decreases, metabolism is enhanced, and these mutant mice obtain a certain resistance to obesity caused by high‐fat diet.^277^ Besides, autophagy can also influence the obesogenic phenotype through inflammatory reactions.^278^, ^279^ An in‐depth investigation regarding the specific role of autophagy in the pathogenesis of obesity is necessary in order to fully exploit its therapeutic potential in prevention and treatment of obesity.

#### 3.3.2 Type 2 diabetes

**FIGURE 4:**
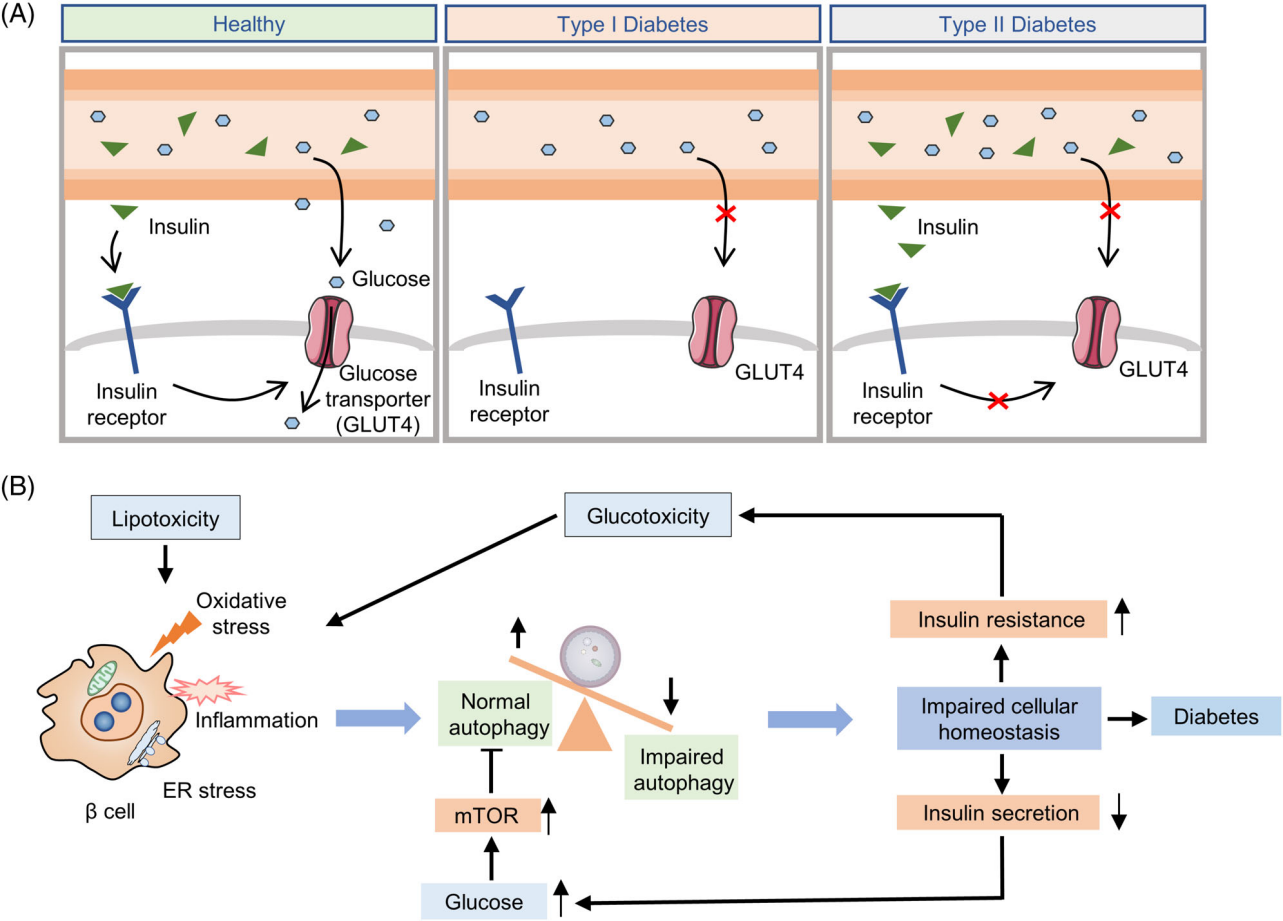
Pathogenesis of diabetes and the implication of autophagy in diabetes. (A) The schematic diagram of type I diabetes and type II diabetes; (B) the effects of insulin resistance and insulin secretion on β‐cell autophagy. Various stress and inflammatory responses to lipotoxicity can induce polyubiquitination of proteins and cytoplasmic aggregation of damaged organelles in β‐cells. Impaired autophagy leads to cellular dysfunction, affects insulin secretion, results in insulin resistance, and promotes diabetes progression

T2D clinically manifests with the appearance of insulin resistance and relative insulin deficiency (Figure 4A). Oxidative stress, inflammation, ER stress, and mitochondrial dysfunction in pancreatic β‐cells, liver, skeletal muscle, and adipose tissue are closely related to the pathogenesis of T2D.^268^ In addition, aging is also a recognized risk factor for T2D. Notably, autophagy appears to have etiological significance (Figure 4B). On the one hand, autophagy‐deficient and insulin‐responsive tissues fail to effectively alleviate oxidative stress and ER stress caused by persistent stimulation of insulin.^271^, ^280^ On the other hand, the homeostatic functions of pancreatic β‐cells require the participation of autophagy.^281^, ^282^ It has been demonstrated that genetic ablation of *Atg7* in β‐cells results in islet degeneration and impaired glucose tolerance, accompanied by reduced insulin secretion, suggesting that autophagy is necessary to maintain the structure, mass, and function of pancreatic β‐cells.^281^, ^282^ Besides, Yamamoto et al.^280^ revealed that autophagy hyperactivation enhances insulin signaling, but the insulin storage and secretion as well as glucose tolerance could be reduced by selective sequestration and degradation of insulin granule vesicles by autophagy in β‐cells. Unlike most cell types, short‐term starvation in pancreatic β‐cells triggers nascent granule degradation and Golgi membrane‐associated degradation mechanisms and it inhibits autophagy via activation of MTOR, thereby inhibiting insulin secretion and preventing hypoglycemia under physiological starvation conditions.^283^, ^284^

Given the established role of autophagy in maintaining β‐cells homeostasis under stressful conditions, intervention in autophagy early in the development of diabetes may help patients retain β‐cells mass and prevent disease progression.^285^ Recently, Cheng et al. found that a simulated fasting diet promoted β‐cells regeneration in a mouse model of T2D. This effect can be mimicked by simultaneous inhibition of the autophagy negative regulators MTORC1 and PKA (protein kinase A), suggesting that autophagy may play a role in islet reprogramming.^286^ Indeed, mimicking glycolipid toxicity in cultured β cells can inhibit autophagy by reducing lysosome‐related gene expression, impairing autophagosome–lysosomal fusion, or reducing lysosomal acidification.^287^, ^288^, ^289^ Furthermore, activated autophagy under stress conditions and blockade of autophagic flux leads to the accumulation of defective lysosomes, causing β‐cells death.^290^ Metformin, rosiglitazone and GLP‐1 mimetics are highly effective drugs for treatment of diabetes and have been shown to effectively induce autophagy in β cells.^291^ As a key inhibitor of autophagy, a large number of studies have been conducted to explore whether MTORC1 can be inhibited as a strategy for diabetes treatment.^292^, ^293^, ^294^ Overall, caution and thoughtful design should be taken into consideration for diabetes therapies via targeting autophagy. Further studies are needed to elucidate the mechanisms of autophagy in cell survival and apoptosis to develop therapeutics that can selectively promote β‐cells survival.

### 3.4 Autophagy in viral infection

One key aspect of the biological functions of autophagy is its regulatory role in pathogen infection, inflammation, and immunity. So far, the relationship between autophagy and infection has been extensively studied and there are rather complicated relationships between these two important processes.^295^, ^296^ First, autophagy can target invaded pathogens such as bacteria and viruses for clearance. Autophagy thus serves as a potent anti‐infection and anti‐inflammatory mechanism. Second, certain strains of microbes can make use of the autophagy machinery or process to survive, replicate and resist to the immunity. Autophagy thus acts as a proinfection and proinflammatory mechanism. Third, reciprocally invaded microbes have diverse impacts on the autophagy process in the host cells, either promotes or inhibits autophagy in a context‐dependent manner. Fourth, autophagy can directly or indirectly regulate both innate and adaptive immunity and the associated inflammatory processes. And finally, targeting autophagy (either promoting or inhibiting) has been an attractive approach in treatment of various forms of infections and related infectious diseases.^297^, ^298^, ^299^

At present, all of the above points have been extensively discussed in literature. In this review, we will mainly focus on the implication of autophagy in coronavirus disease of 2019 (COVID‐19) caused by SARS‐CoV‐2. Since the outbreak at the end of 2019, SARS‐CoV‐2 has quickly spread to the whole globe, causing a major pandemic with severe loss to both life and economy. Based on WHO (https://covid19.who.int/), up to date (April 20, 2022), there are more than 500 million confirmed cases, with more than 6.2 million deaths worldwide. Despite the enormous efforts in control of this pandemic, including vaccination and lockdown of major cities around the world, there is no clear sign of control, especially with the emerging variants with increased transmissibility and pathogenicity.^300^, ^301^

**FIGURE 5:**
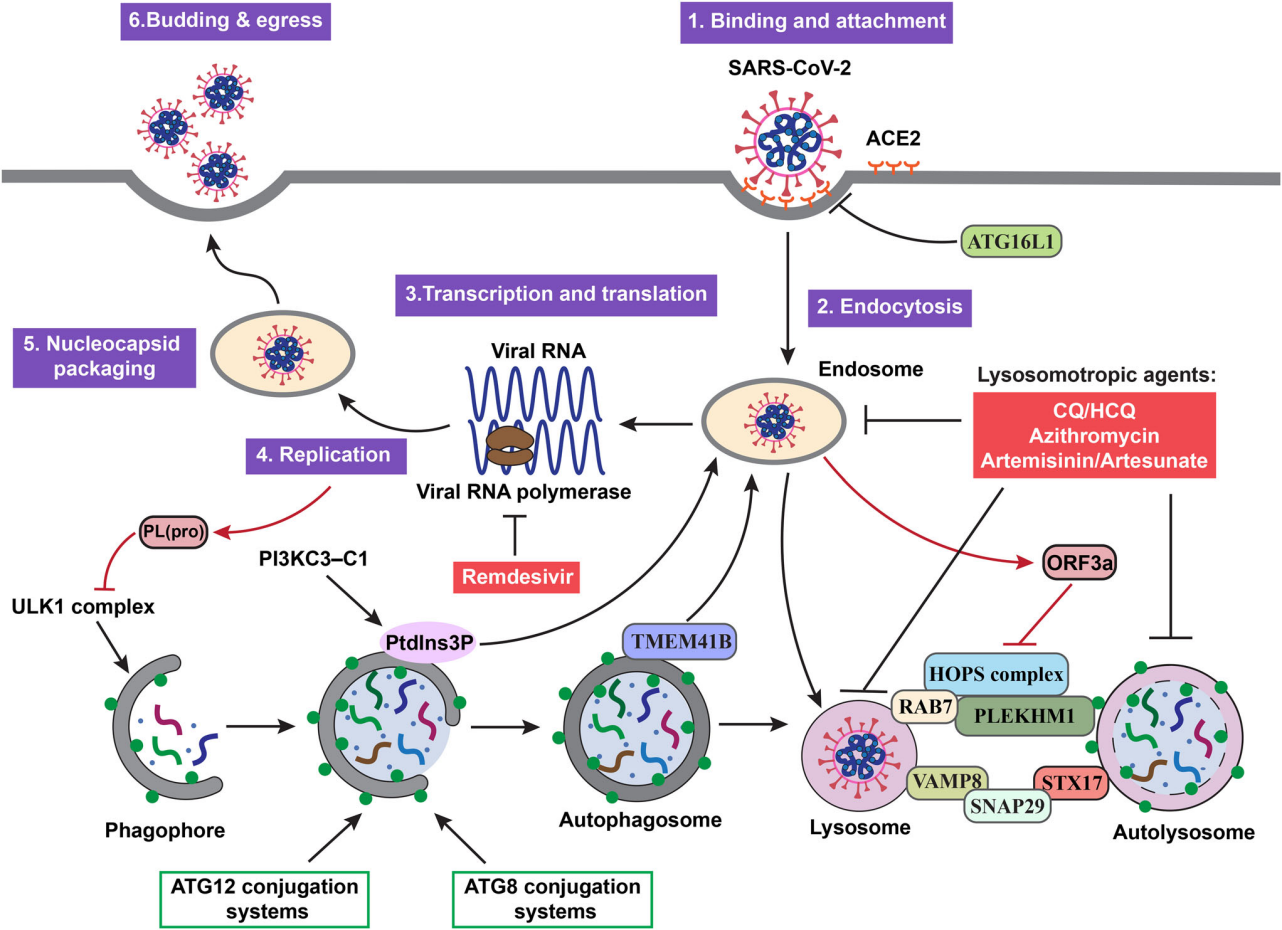
Targeting the autophagy‐lysosome pathway as novel therapeutic strategies for COVID‐19. The replication cycle of SARS‐CoV‐2 consists of six consecutive steps, as well as the cross‐talks of this cycle with the autophagy–lysosome pathway are presented. The red boxes indicate that the lysosomotropic agents targeting the autophagy–lysosome pathway are under development as therapeutics against COVID‐19

Biologically, SARS‐CoV‐2 is a type of RNA viruses with a positive‐sense single‐stranded RNA genome. Its genome encodes 11 genes with 14 open reading frames (ORFs) that produce 16 nonstructural proteins, four structural proteins, and nine accessory proteins.^302^ As shown in Figure 5, the replication process of SARS‐CoV‐2 has also been well established, consisting of the following six steps^303^, ^304^: (i) binding: the virus first binds to the host cell surface via the interaction between the viral S protein and host receptor angiotensin‐converting enzyme‐2 (ACE2); (ii) endocytosis: the virus enters into the host cell's endocytic pathway (endosomes and lysosomes) via fusion of viral membrane with the host cell membrane; (iii) transcription and translation of viral proteins from the viral RNAs; (iv) viral replication within the replicative membranous compartment; (v) nucleocapsid packaging: virus assembly and package at the ER and/or the Golgi complex; and finally (vi) budding and egress: release of new virions via exocytosis.

One important research topic in the study of SARS‐CoV‐2 and COVID‐19 is the role of the autophagy and lysosome system in the pathogenicity of this deadly disease. Up to date, there is accumulating evidence demonstrating the reciprocal nature of interaction between autophagy and SARS‐CoV‐2 and COVID‐19.^305^, ^306^, ^307^, ^308^ Here, we will provide an update on the effect of autophagy on SARS‐CoV‐2 and COVID‐19 and the reciprocal effects of SARS‐CoV‐2 on autophagy. Understanding such intricate relationship is important for development of novel therapeutic approaches of COVID‐19 by targeting the autophagy pathway.

#### 3.4.1 The effects of autophagy on SARS‐CoV‐2 and COVID‐19

Similar to the effects of autophagy on many other types of viruses, autophagy also has dual effects against SARS‐CoV‐2: proviral and antiviral effects. At present, there are multiple lines of evidence suggesting that the autophagy machinery or the autophagy process can assist SARS‐CoV‐2 in the cycle of viral replication as highlighted in Figure 5.

First, ACE2 is the receptor at the host cell's plasma membrane that is able to directly bind to the receptor‐binding domain of the spike protein of SARS‐CoV‐2.^309^, ^310^ Two recent studies independently identified the presence of LIR motifs in the tails of ACE2,^311^, ^312^ indicating the possible involvement of LC3 in the initial step of viral infection, although the exact significance of this LIR motifs in the viral infection remains to be further determined.

Second, mechanistically, there are novel molecular links connecting autophagy with SARS‐CoV‐2 replication. For instance, by using genome‐wide CRISPR‐Cas9 screening, TMEM41B (transmembrane protein 41B), an ER‐localized transmembrane protein, has been identified as a novel autophagy regulator required for autophagosome formation.^313^, ^314^, ^315^ Interestingly, two recent studies demonstrated that TMEM41B is required for SARS‐CoV‐2 infection, based on the observations that deletion of *TMEM41B* markedly reduced infectivity, which could be fully restored with the reconstitution of *TMEM41B*.^316^, ^317^ With increasing research on SARS‐CoV‐2 and autophagy, we expect to see more such common regulators to be discovered.

Third, although the canonical autophagy proteins such as BECN1, ATG5, and ATG7 are not required for viral infection of SARS‐CoV‐2 in the host cells,^316^ the class III PI3K (phosphoinositide 3‐kinase) or PIK3C3, a key regulator in autophagy, has been shown to play an important role in viral replication of SARS‐CoV‐2: (i) PI3K is activated in cells infected with SARS‐CoV‐2 to produce PtdIns3P^318^; (ii) inhibition of PI3K kinase activity led to suppression of SARS‐CoV‐2 replication in human airway epithelial cells.^319^ Intriguingly, other autophagy inhibitors such as ULK1 inhibitor SBI0206965 and lysosome inhibitor hydroxyl chloroquine (HCQ) failed to exert similar inhibitory effects on SARS‐CoV‐2 replication, suggesting the possibility that it is PIK3C3, but not autophagy per se that may participate the replication process of SARS‐CoV‐2.^320^ Obviously more work is needed to establish the therapeutic value of targeting the autophagy machinery in treating COVID‐19.

At present, relatively little is known about the possible antiviral effect of autophagy against SARS‐CoV‐2. ATG16L1 is one of the key ATGs in control of autophagosome biogenesis by forming a protein complex with ATG12–ATG5 in mediating LC3 lipidation.^321^ In response to SARS‐CoV‐2 infection, its receptor ACE2 is able to recruit ATG16L1 to form exosome‐like vesicles called defensosomes that are capable of blocking viral entry.^322^ Moreover, it is known that an ATG16L1 allele (ATG16L1‐T300A) was associated with impaired autophagy/xenophagy and reduced ability in clearing pathogens.^323^ Interestingly, people bearing the ATG16L1 T300 allele expressed higher level of ACE2 compared with ATG16L1 A300, suggesting that ATG16L1 T300 is associated with a greater probability of SARS‐CoV‐2 infection,^324^ although the mechanisms linking ATG16L1 and ACE2 remains unknown.

#### 3.4.2 The reciprocal effects of SARS‐CoV‐2 on autophagy

What we have discussed above is the effect of autophagy on SARS‐CoV‐2. Interestingly, there is accumulating evidence suggesting that SARS‐CoV‐2 has reciprocal effect on autophagy, either activate or suppress via a variety of mechanisms. For instance, papain‐like protease PL(pro), a viral protease from SARS‐CoV‐2, is able to disrupt formation of ULK1–ATG13 complex and eventually suppress starvation‐induced autophagy at the early stage of autophagosome biogenesis^325^ (Figure 5).

However, majority of the evidence on the inhibitory effect of SARS‐CoV‐2 on autophagy is actually on the late stage, especially on fusion of autophagosome with lysosome, a process known to be controlled by a group of protein complexes including SNARE (STX17–SNAP29–VAMP8), HOPS (VPS39, VPS11), and ATGs (ATG14).^96^, ^97^ Multiple studies have pointed to ORF3a, an accessory viral protein from SARS‐CoV‐2 that is capable of suppressing the autophagic flux by disrupting autophagosome–lysosome fusion.^326^, ^327^, ^328^, ^329^, ^330^ Mechanistically, ORF3a is able to interact with VPS39, an important component of the HOPS complex in control of autophagosome–lysosome fusion.^331^, ^332^ On the other hand, ORF3a is also capable of inhibiting autophagy and promoting lysosomal exocytosis and extracellular egress of SARS‐CoV‐2.^333^

Moreover, another viral protein from SARS‐CoV‐2, NSP6, has also been found to impair the autophagic flux at the late stage by targeting ATP6AP1 (ATPase H+ transporting accessory protein 1), a key component of the lysosomal V‐ATPase in control of lysosomal acidity.^334^ Similarly, SARS‐CoV‐2 and other CoVs such as MHV are enriched in the endocytic organelles including endosomes and lysosomes, leading to lysosome deacidification and suppression of autophagy at the late stage.^335^

In addition to the inhibitory effects on autophagy by SARS‐CoV‐2, there are reports demonstrating the opposite effects on autophagy. For instance, infection with SARS‐CoV‐2 enhanced LAMP2a expression and upregulated autophagic flux.^336^ The autophagy‐promoting activity of SARS‐CoV‐2 has also been demonstrated in several animal models in vivo, including human ACE2 transgenic mice.^337^ Such results thus provide experimental basis for using autophagy inhibitors including CQ for treatment of COVID‐19, a topic to be discussed in the section below.

#### 3.4.3 Targeting autophagy as a potential therapeutic strategy for treatment of COVID‐19

Since the outbreak of COVID‐19 more than 2 years ago, the efforts in developing effective therapeutic drugs for clinical application never stop, although with only limited success, in comparison with the much more successful vaccination program. The initiation excitement with Remdesivir did not last that long. Remdesivir was approved by United States Food and Drug Administration (US FDA) while WHO provide the opposite advice on its clinical application in treatment of COVID‐19, mainly due to its inconsistent and controversial clinical outcomes.^338^, ^339^, ^340^, ^341^ In December 2021, another oral medication PAXLOVID (nirmatrelvir + ritonavir, inhibitors of viral *proteases*) has received US FDA's emergency use authorization for COVID‐19.

Among many types of antiviral drugs tested against COVID‐19 so far, the lysosomotropic agents (CQ/HCQ) have received much attention.^342^ Pharmacologically, both CQ and HCQ are with lysosomotropic property, capable of enriching inside the endolysosomes and chemically neutralizing the pH, leading to suppression of the degradative function of the endolysosomal system. Based on this unique pharmacological activity, CQ/HCQ has a long history of treating malaria and also has been widely used as autophagy inhibitors by suppressing lysosomal function.^343^, ^344^ In fact, CQ/HCQ are the only autophagy inhibitors in clinical trials in autophagy‐related diseases such as cancer.^345^ Based on the existing knowledge of the close implication of the autophagy‐lysosome system in the replication cycle of SARS‐CoV‐2,^306^ CQ/HCQ quickly caught people's attention as potential therapeutic agents against COVID‐19. Unfortunately, despite the strong evidence from preclinical studies (cell culture and animal models), the outcomes of many clinical trials using CQ/HCQ are largely disappointing, accompanied by significant side effects.^346^, ^347^, ^348^ Thus, US FDA revoked its authorization for the emergency use of CQ/HCQ in COVID‐19 patients (https://www.fda.gov/news‐events/press‐announcements/coronavirus‐covid‐19‐update‐fda‐revokes‐emergency‐use‐authorization‐chloroquine‐and).

In addition to CQ/HCQ, several other lysosomotropic agents have been investigated. One example is azithromycin, a commonly used antibiotic that has similar lysosomotropic property as CQ/HCQ and this pharmacology effect is related to its antibacterial and antiviral function.^349^, ^350^ Recent work has indicated that azithromycin is able to block the entry of SARS‐CoV‐2 via disruption of the fusion process between viral and vacuolar membranes, a process related to its lysosomotropic property.^351^ Moreover, numerous clinical trials have been conducted, using either azithromycin alone or in combination with CQ/HCQ in treatment of COVID‐19.^352^, ^353^, ^354^, ^355^, ^356^ Despite the therapeutic benefits of using azithromycin alone or in combination with CQ/HCQ in treatment of COVID‐19, more work is needed to move azithromycin from bench to bed.

Artemisinin and its related compounds from the extracts of the medicinal plant, *Artemisia annua* L., have been widely used as an antimalarial drug and the main researcher of this wonder drug, Youyou Tu, won the Nobel Prize in Physiology or Medicine in 2015. Interestingly, it has been reported that artesunate, a derivative of artemisinin, has lysosomotropic property and is able to activate lysosomal function.^357^ At present, there is evidence demonstrating the antiviral activity of artemisinin and some of its derivatives such as artesunate, artemether, and dihydroartemisinin against SARS‐CoV‐2, from in vitro cell‐based assays, to animal models and clinical trials, either alone or in combination with other therapeutic agents.^358^, ^359^, ^360^ Understanding the potential therapeutic of artemisinin and its derivatives in COVID‐19 is important, especially for control of this pandemic in less‐developed countries and regions such as Africa where malaria and COVID‐19 constitute two major public health challenges.^361^, ^362^

### 3.5 Autophagy in cardiovascular diseases

**FIGURE 6:**
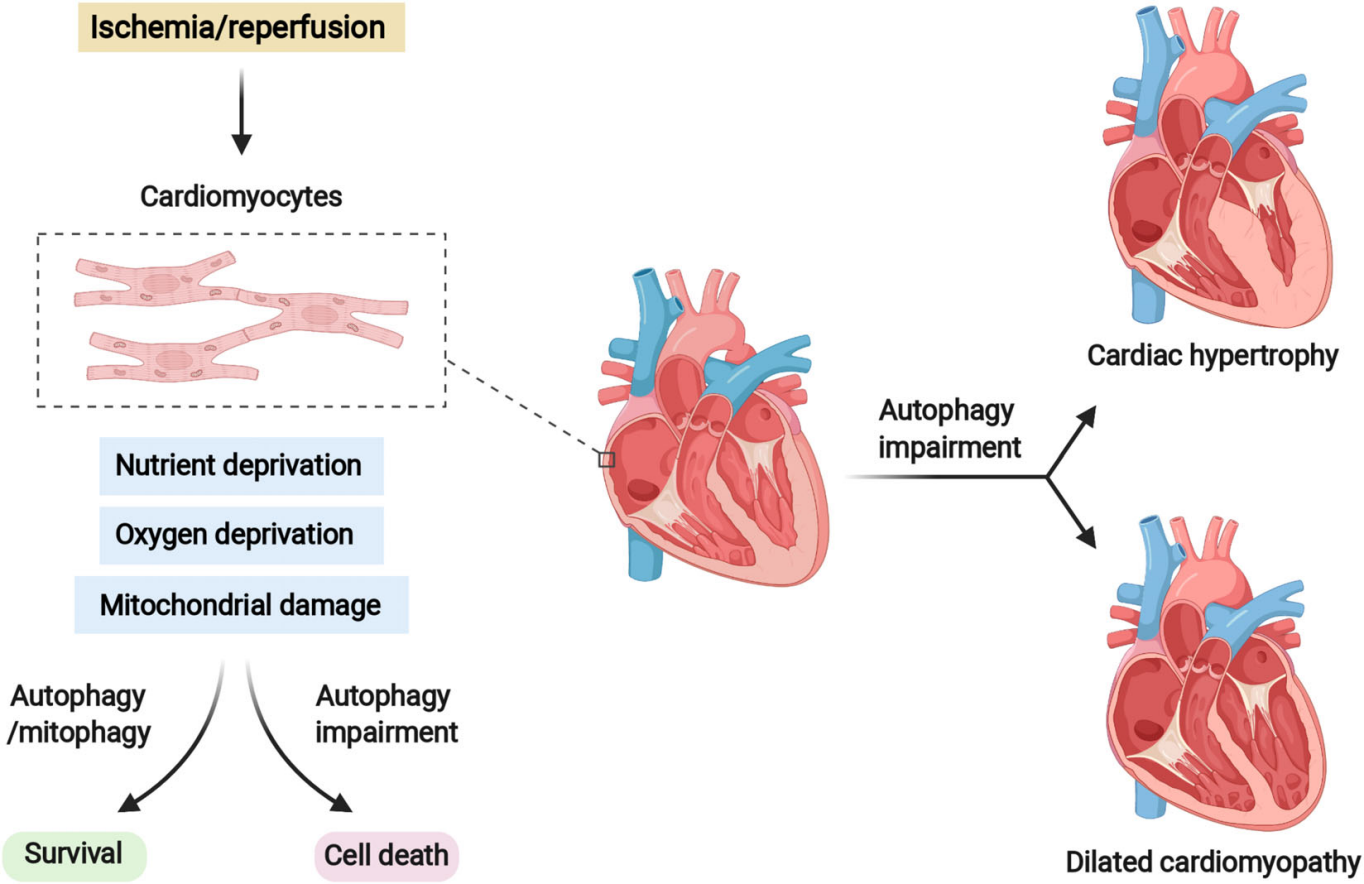
The protective effects of autophagy on cardiovascular diseases. Autophagy is essential for maintaining the intracellular homeostasis of cardiomyocytes under both basal and stress conditions. In response to cardiac ischemia/reperfusion, autophagy acts as a survival mechanism for replenishment of metabolic substrates and removal of damaged organelle such as damaged mitochondria. Impairment of autophagy results in cardiomyocyte cell death and exacerbates ischemia/reperfusion injury. Dysregulation of autophagy is also related to other cardiovascular diseases such as cardiac hypertrophy and dilated cardiomyopathy

Cardiovascular disease is the leading cause of death in the developed world.^363^ Increasing number of studies have linked autophagy to cardiovascular health and disease (Figure 6). Generally, basal autophagy plays a vital role in maintaining the intracellular homeostasis of the major cardiovascular cell types, including cardiomyocytes, cardiac fibroblasts, endothelial cells, vascular smooth muscle cells, and macrophages via clearance of superfluous or damaged cellular components.^364^ Moreover, autophagy is also crucial for cardiac progenitor cell differentiation.^365^, ^366^ It has been further shown that depletion of essential *ATGs* impairs cardiac morphogenesis.^367^ These findings collectively reflect the beneficial effects of autophagy on cardiovascular health and thus dysregulation of autophagy is believed to play a role in the pathogenesis of various cardiovascular disorders.^368^ Here, we mainly focus on the roles of autophagy in cardiac ischemia/reperfusion injury, cardiac hypertrophy, and dilated cardiomyopathies.

#### 3.5.1 Autophagy in ischemia/reperfusion injury

Ischemic heart disease is one of the leading causes for sudden cardiac death worldwide. During ischemia, the supplies of nutrients and oxygen are limited, which is accompanied by mitochondrial damage.^369^ In response to ischemia, AMPK is activated, leading to suppression of MTORC1 and hence activation of autophagy.^370^ Functionally, cardiomyocyte autophagy acts as a cytoprotective mechanism for replenishment of metabolic substrates and removal of damaged organelle such as mitochondria.^371^ Accordingly, enhancing autophagy via activating AMPK has been shown to protect against ischemic injury,^372^, ^373^, ^374^ while suppression of autophagy promotes cardiomyocyte cell death.^375^ As damaged mitochondria are responsible for releasing ROS, selective removal of damaged mitochondria by mitophagy has been proposed as a key mechanism to protect against ischemia injury.^376^, ^377^ Consistently, loss of Parkin or Pink1, the two key players in mitophagy, increases the heart's vulnerability to ischemic injury in mouse model.^378^, ^379^

During reperfusion, the level of nutrients and oxygen are restored to normal level. Intriguingly, it has been reported that autophagy is still activated during reperfusion via an AMPK–MTORC1‐independent mechanism.^370^ However, whether autophagy is beneficial or detrimental under such condition is a subject of debate, with no consensus reached so far. On one hand, it has been shown that reperfusion injury is blunted by activation of autophagy flux,^380^, ^381^, ^382^ suggestive of the protective effect of autophagy. Of note, the cytoprotective effects of autophagy on cardiomyocyte involves the suppression of ROS production.^380^ Consistently, activation of mitophagy also attenuates ROS production and cardiomyocyte cell death in response to reperfusion.^383^ Impaired autophagy contributes to cardiomyocyte cell death and exacerbates reperfusion injury.^384^, ^385^, ^386^ On the other hand, there is evidence suggesting that autophagic cell death is involved in reperfusion injury^387^ and pharmacologic inhibition of excessive autophagy protects against myocardial reperfusion injury.^375^, ^388^ Thus, further studies are necessary to define whether increased autophagy in response to reperfusion is an epiphenomenon or a causative factor in cardiomyocyte cell death.

#### 3.5.2 Autophagy in cardiac hypertrophy

Cardiac hypertrophy refers to the abnormal enlargement or thickening of the heart muscle, which is defined as an adaptive response and accompanied by other cardiovascular diseases such as hypertension, ischemic disease, and heart failure (HF).^389^ The first study on cardiac hypertrophy and autophagy dated 1983, in which reduced autophagic vacuoles were observed in ventricular hypertrophy derived from supravalvular aortic constriction.^390^ Cardiac‐specific deficiency of *Atg5* reveals the importance of autophagy in maintaining cardiomyocyte size and global cardiac structure and function.^391^ Moreover, in adult mice, temporally controlled cardiac‐specific deficiency of *Atg5* led to various cardiomyopathies including cardiac hypertrophy, left ventricular dilatation, and contractile dysfunction.^391^ Mutations of *LAMP2* were also reported in association with severe cardiac hypertrophy.^392^ Deficiency of Foxo32 (also known as MAFbx or Atrogin‐1), a muscle‐specific gene required for muscle atrophy, impairs autophagic flux, and results in heart hypertrophy in mice.^393^

As the key mechanism to remove dysfunctional mitochondria to ensure cardiomyocyte energetic and metabolic needs, mitophagy also plays a pivotal role in preventing cardiac hypertrophy.^394^ Mice with Parkin or Pink1 depletion exhibit enhanced cardiac hypertrophy and contractile dysfunction in response to pressure overload.^395^ It has also been reported that mice deficient in Pink1 develop early left ventricular dysfunction and pathological cardiac hypertrophy.^396^ Intriguingly, BNIP3, a key player involved in Parkin‐independent mitophagy, appears to accelerate the progression of cardiac hypertrophy.^397^ Given that BNIP3 is also involved in other cellular processes such as inflammation, apoptosis, and necrosis,^398^ BNIP3‐mediated mitophagy could be an epiphenomenon associated with cardiac hypertrophy progression. Further study is necessary to determine whether BNIP3‐mediated mitophagy is a compensatory mechanism to sustain the heart function in the course of cardiac hypertrophy.

Misfolded proteins and intracellular aggregates have been shown to contribute to cardiac hypertrophy.^399^ One good example is the small heat shock protein alphaB‐crystallin (CryAB) whose missense mutation (CryAB^R120G^) causes aberrant Desmin and CryAB aggregation and results in cardiac hypertrophy.^400^ At cellular level, it has been shown that CryAB^R120G^ triggers ATG7‐depedent autophagy, leading to clearance of CryAB^R120G^ aggregates in cardiomyocytes.^401^, ^402^ Similarly, Becn1 haploinsufficiency results in reduction of autophagy and promotes HF progression in αMHC‐CryABR^120G^ mice.^401^

The above‐mentioned findings collectively connect impaired autophagy to cardiac hypertrophy. Activation of autophagy at the whole‐body levels by nutritional (such as caloric restriction [CR] or spermidine that operates as a CR mimetic [CRM]) or pharmacological (such as rapamycin) interventions shows beneficial effects on aged‐dependent cardiac hypertrophy reversal.^403^

#### 3.5.3 Autophagy in dilated cardiomyopathy

Dilated cardiomyopathy (DCM) refers to a syndrome manifested by cardiac enlargement and poor systolic function of the left ventricular, with an ejection fraction <45%, which is a major cause for HF.^404^ The evidence of autophagy in heart diseases was first reported in tissue samples from DCM patients.^405^ However, the exact role of autophagy in DCM in this study was not studied. A more recent study shows that the autophagic vacuoles are identified in the cardiomyocytes with myofilament changes in the left ventricular.^406^ Importantly, the number of autophagic vacuoles in cardiomyocytes is associated with a better HF prognosis in patients with DCM,^406^ suggesting the importance of autophagy in prevention of HF resulted from DCM. Conversely, mice with cardiac‐specific deficiency of *Atg5* develop left ventricular dilatation and contractile dysfunction.^391^ A mutation in the *LAMP2* gene (c.928G>A mutation) previously found in patients with hypertrophic cardiomyopathy is shown to be associated with severe DCM.^407^ Moreover, CryABR^120G^ has also been reported in DCM. In mice model, expression of CryABR^120G^ results in aberrant mitochondrial‐sarcomere architecture and defective mitochondrial function, leading to apoptosis of cardiomyocyte and eventually DCM and HF.^408^ Given the importance of autophagy in clearance of protein aggregates, it is believed that autophagy possesses beneficial effects in prevention of DCM.

### 3.6 Autophagy in aging

**FIGURE 7:**
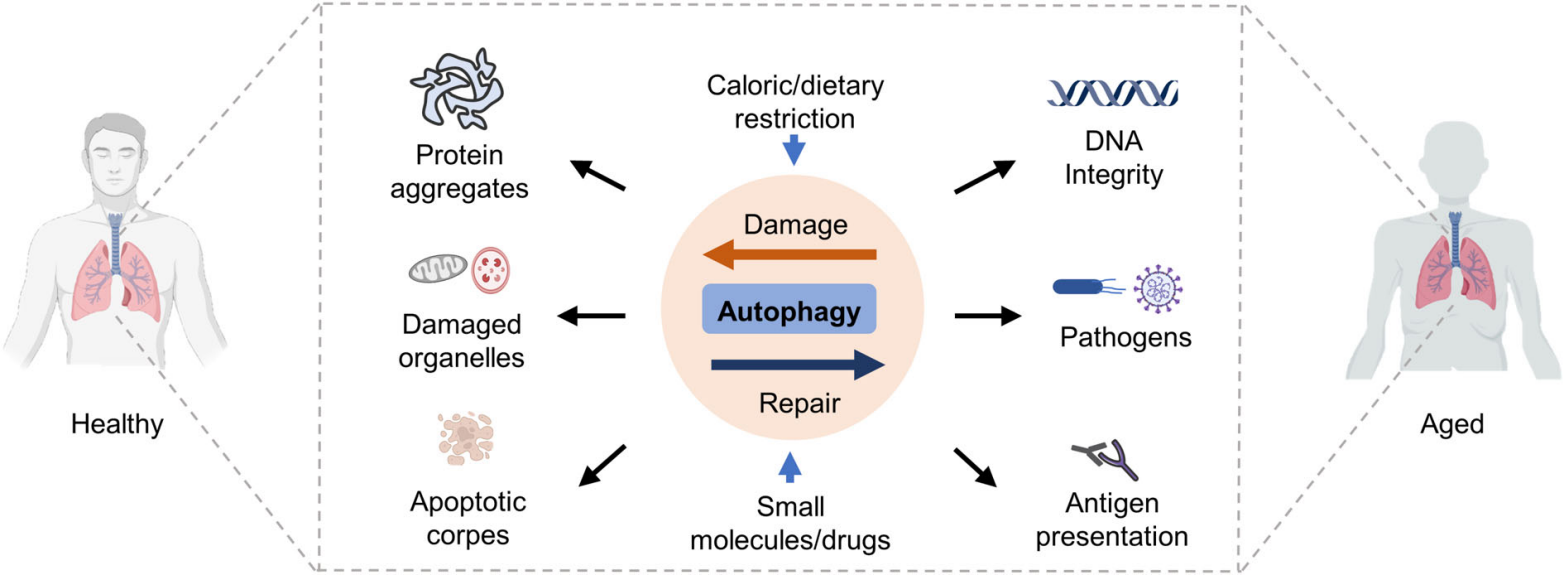
Systemic antiaging effects of autophagy. Autophagy may increase organismal fitness by eliminating protein aggregates, damaged organelles, apoptotic corpses. In addition, autophagy may contribute to the clearance of intracellular pathogens and enhancing the function of antigen‐presenting cells to attenuate age‐related dysfunctions and extend lifespan

Aging is a biological process characterized by time‐dependent cellular and functional decline and a main risk factor associated with the progress of many disorders such as cancer, cardiovascular diseases, and neurodegenerative diseases, leading to reduced life quality of the organisms.^409^ Diverse stress response pathways, including autophagy, promote growth and delay aging by limiting tissue damage, promoting tissue repair and alleviating environmental pressures.^410^, ^411^ Despite the complex relationship between autophagy and aging, a growing body of research supports the critical role of autophagy in promoting longevity and delaying aging (Figure 7). As a conserved degradation pathway, autophagy promotes cell survival by removing aged molecules and mobilizing energy substances.^412^ Impaired autophagy causes diverse cellular dysfunctions that predispose individuals to age‐related diseases and exacerbate the aging process, whereas enhanced autophagy prolongs lifespan and improves health span.^413^ Therefore, an in‐depth understanding of the cellular biological functions of autophagy in aging is critical for developing autophagy‐targeted therapies for aging and aging‐related diseases.

One important observation in autophagy and aging study is that aging is accompanied by reduced lysosomal protease activity and reduced expression of several ATGs in rodent and Drosophila.^414^, ^415^, ^416^ Similarly, in *C. elegans*, the autophagy activity generally declines with aging, whereas long‐lived mutants of Daf‐2 and Glp‐1 regulate autophagy in different spatiotemporally specific ways to prolong lifespan.^417^ In drosophila, loss‐of‐function mutations of Atg7 or Atg8 promote the accumulation of ubiquitin‐positive aggregates in neurons and increase sensitivity to stress, thereby shortening the drosophila lifespan.^415^, ^418^ In mice, disruption of the BECN1–BCL2 complex is an effective mechanism for enhancing autophagy, preventing premature aging, promoting longevity, and improving health.^419^ In humans, the expression levels of ATG5, ATG7, and BECN1 are downregulated with aging.^420^ These studies collectively suggest anti‐aging as a primordial function of autophagy.

Among the various signaling networks regulating autophagy, the roles of MTOR and AMPK in regulation of aging and lifespan attract particular attention.^413^ Upregulation of TOR signaling and insulin/IGF‐1 (IIS) pathway has been widely reported during aging, and inhibition of TOR signaling is sufficient to promote longevity in an autophagy‐dependent manner.^421^ Similarly, inhibition of AMPK activity also occurs during aging, and its activation prolongs the lifespan of *C. elegans* by elevating autophagy.^422^ Given the critical roles of MTOR and AMPK in control of nutrient metabolism, CR without malnutrition is an effective intervention to delay aging and prevent aging‐related diseases in most species and autophagy is likely to be one of the underlying mechanisms, whereas autophagy inhibition diminishes the life‐extending effects of CR.^423^, ^424^, ^425^ CR‐induced autophagy may involve multiple mechanisms, mainly via inhibiting MTOR signaling. Consistently, CR‐like lifespan extension can be achieved with the MTOR inhibitor rapamycin.^426^ In addition, rapamycin may extend lifespan by inhibiting inflammation and reducing protein imbalances that lead to aging.^427^, ^428^ These results suggest that MTOR inhibitors can be used to treat age‐related diseases, but their side effects, such as immunosuppression and insulin resistance, limit their widespread application as an anti‐aging therapy.^427^ This has driven the development of MTOR inhibitors with superior pharmacodynamics and agents that mimic the effects of CR, the so‐called CRMs, such as metformin, spermidine, and resveratrol.^413^ Applying precision medicine for drug adaptation and artificial intelligence‐based dosing strategies to promote longevity by enhancing autophagy is thus promising.

## 4 MODULATION OF AUTOPHAGY AS THERAPEUTIC STRATEGY IN HUMAN DISEASES

**FIGURE 8:**
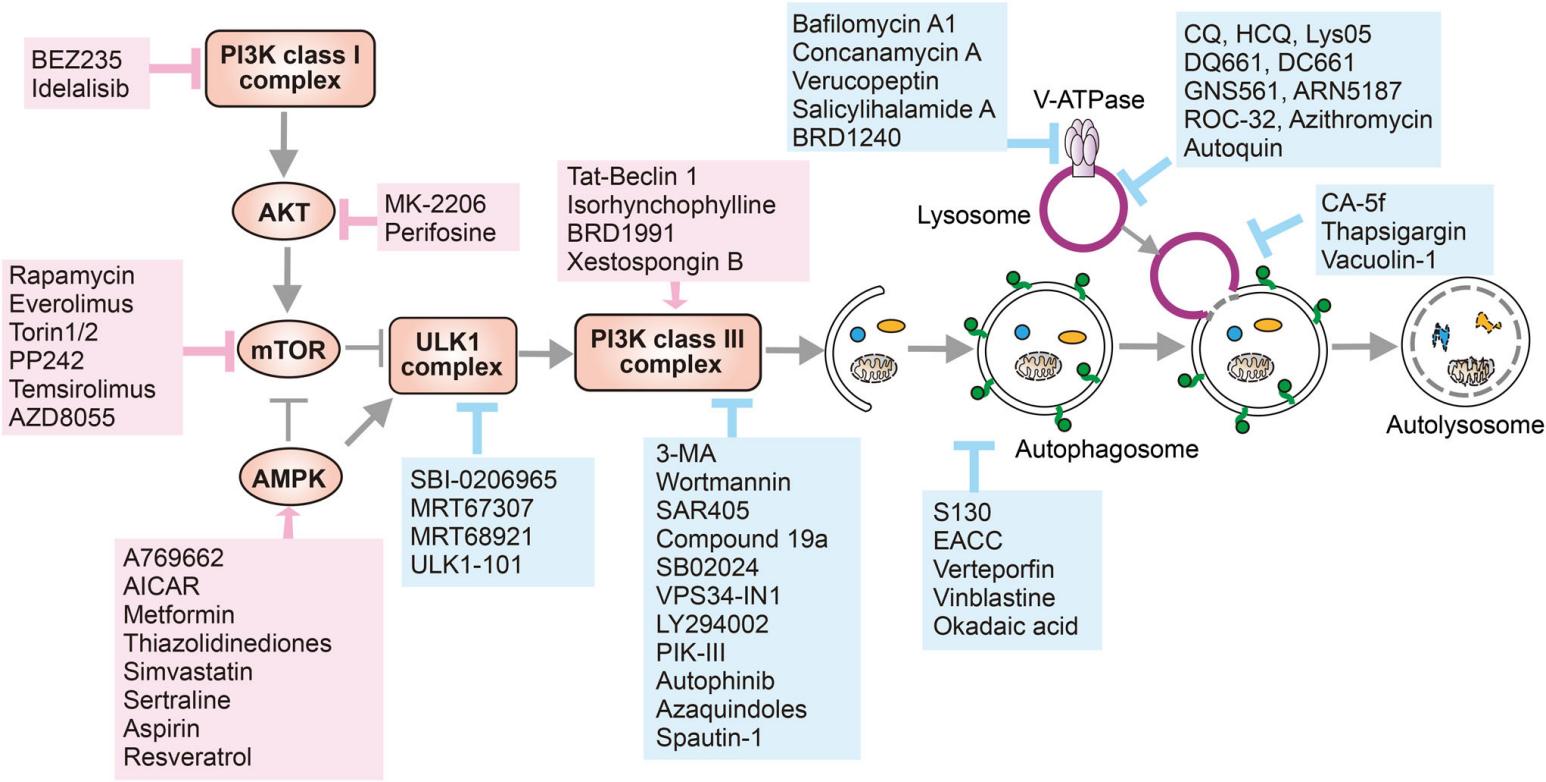
Autophagy activators and inhibitors. Mechanistic targets of autophagy activators and inhibitors. The major targets and related compounds of autophagy activation (arrows and boxes in red) and inhibition (arrows and boxes in blue) have been demonstrated

As we discussed in the previous section, approaches modulating autophagy are demanded by its important role in human health and disease. Based on the intention of different clinical applications, both autophagy activators and inhibitors have been developed and tested in the past several decades (Figure 8). Here, we reviewed and grouped the known autophagy modulators in this section.

### 4.1 Activation of autophagy

Activation of autophagy has been mainly achieved via activation of AMPK or/and suppression of PIK3CA/AKT/MTOR signaling (Table 2).

**TABLE 2:** Known autophagy pharmacological activators and their potential application in disease

Class	Agent	Mechanism of action	Biological effects and application in disease	References
AMPK activator	A769662	Allosterically activating AMPK	Type‐II diabetes and associated metabolic disorders (preclinical)	^429^, ^430^
	AICAR	Allosterically activating AMPK via its metabolite ZMP, which is an AMP mimetic	Type‐II diabetes (clinical trials); intestinal barrier function and epithelial differentiation (preclinical)	^431^, ^432^
	Metformin	Inhibiting RCC1 (respiratory chain complex‐1) to raise the cellular ATP/AMP ratio; upregulating of SIRT1 expression	First‐line antidiabetic drug (approved); potential application of in cancer treatment (clinical trials), including osteosarcoma, prostate cancer, and so on	^433^, ^434^, ^435^
	Thiazolidinediones	Inhibiting RCC1, to raise the cellular ATP/AMP ratio	Potential application of in cancer treatment (preclinical); diabetes, obesity (clinical trials)	^436^, ^437^
	Simvastatin	Raising cellular ATP/AMP ratio via inhibiting cholesterol biosynthesis	Antihypercholesterolaemia (approved); cardioprotective effects (approved); potential application of in cancer treatment (clinical trials)	^438^, ^439^, ^440^
	Sertraline	Antagonizing the mitochondrial VDAC1 (voltage‐dependent anion channel 1), resulting in reduced cellular ATP level	Antidepressant drug (approved)	^441^
	Resveratrol	Enhancing CamKKβ‐AMPK; SIRT1 signaling; upregulating cytoplasmic (de)acetylation reactions	Antioxidant (over‐the‐counter (OTC) supplements); antiaging effects, including anti‐inflammatory, cancer prevention, antidiabetic, cardioprotective, and neuroprotective properties (clinical trials)	^442^, ^443^, ^444^, ^445^, ^446^, ^447^, ^448^
MTORC1 inhibitor	Rapamycin and rapalogs (Sirolimus)	Destabilization of MTOR–RAPTOR complex	Life‐span extension (preclinical studies); to prevent transplant rejection and to treat a rare pulmonary disease (approved); neuroprotective properties (preclinical); anticancer, including breast cancer; advanced pancreatic; non‐small‐cell lung cancer (clinical trials)	^193^, ^449^, ^450^, ^451^
	Everolimus (RAD‐001)	Destabilization of MTOR–RAPTOR complex	Anticancer effects (approved for breast cancer and renal cell carcinoma treatment), kidney transplant failure and rejection	^452^, ^453^, ^454^, ^455^
	Temsirolimus	Destabilization of MTOR–RAPTOR complex	Potential treatment for treatments of LMNA cardiomyopathy, Huntington disease (preclinical); anticancer effects (approved for advanced renal cell carcinoma treatment)	^456^, ^457^, ^458^, ^459^
	Torin1/2	An ATP‐competitive inhibitor for MTOR	Experiment tools	^460^, ^461^, ^462^
	PP242	An ATP‐competitive inhibitor for MTOR	Reduce tumor size in xenograft models (preclinical)	^460^, ^463^, ^464^, ^465^
	AZD8055	An ATP‐competitive inhibitor for MTOR	Advanced solid malignancies (clinical trials)	^188^, ^466^, ^467^, ^468^, ^469^
	EN6	Targeting the ATP6V1A subunit of the lysosomal V‐ATPase, which stimulates MTORC1 via the Rag GTPase	Clears TDP‐43 aggregates, a causative agent in frontotemporal dementia (preclinical)	^470^
AcCoA inhibitor	Hydroxycitrate acid	Competitive inhibiting the ATP citrate lyase (ACLY) to block the AcCoA synthesis, which activates MTOR vis EP300‐induced acetylation	Potential anticancer effects; immunosurveillance (OTC supplements)	^471^
	Triethylenetetramine	A Cu(II)‐selective chelator, which depleting AcCoA via activating of SAT1;	Wilson's disease (approved); corrects diet‐induced metabolic syndrome; prevention or treatment of obesity (preclinical); cancer prevention (clinical trials)	^472^
PI3K and MTOR inhibitor	BEZ235	Binding to the ATP‐binding clefts of PI3K class I and MTOR kinase	Promote survival of drug‐resistant glioma; T‐cell acute lymphoblastic leukemia; hepatocellular carcinoma (clinical trials)	^473^, ^474^, ^475^
PI3K inhibitor	Idelalisib (CAL‐101)	Small‐molecule inhibitor of the delta isoform (p110δ) of PI3K	Chronic lymphocytic leukemia (approved)	^476^, ^477^
AKT inhibitor	MK‐2206	Allosterically inhibiting AKT in a pH‐domain‐dependent inhibitor	Anticancer effect, including colorectal cancer, melanoma, and advanced liver cancer (clinical trials), inhibiting COVID‐19 propagation in vitro (preclinical)	^478^, ^479^, ^480^
	Perifosine	Allosterically inhibiting Akt in a pH‐domain‐dependent inhibitor	Chronic lymphocytic leukemia, small lymphocytic lymphoma (clinical trials)	^481^
Multiple targets (AMPK or MTOR related)	Aspirin (and salicylate)	Activating AMPK; inhibiting EP300, COX (cyclooxygenase)‐1, COX‐2 and MTOR;	Anti‐inflammatory, antimicrobial, antipyretic, and analgesic drug (approved); cardiovascular diseases (clinical trials); anticancer (clinical trials)	^482^, ^483^, ^484^
	Spermidine	Inhibiting acetyltransferases (such as EP300) leading to increased cytoplasmic deacetylation reactions; inhibiting MTOR; activating AMPK	Antioxidant; antiaging effects, including anti‐inflammatory, cancer prevention (clinical trials), antidiabetic, cardioprotective (clinical trials), and neuroprotective properties	^442^, ^471^, ^485^, ^486^, ^487^, ^488^, ^489^, ^490^, ^491^
	EGCG	CaMKKβ–AMPK signaling; MTOR, HATs, and KDACs	Potential therapeutic reagent to prevent cardiovascular complication, metabolic syndromes and potential anticancer effect (clinical trials)	^492^, ^493^
	Curcumin	AMPK‐MTOR; TFEB‐lysosome pathway	Potential anticancer effect (clinical trials)	^494^, ^495^, ^496^
	Trehalose	Inhibition of glucose transporter SLC2A (also known as GLUT) to induce AMPK‐dependent autophagy; low‐grade lysosomal stress‐mediated TFEB activation	Neurodegenerative diseases, cardioprotective effects, atherosclerosis, hepatic steatosis; bipolar disorders, dry eye syndrome and vascular ageing, Alzheimer disease, nonalcoholic fatty live, infertility (clinical trials)	^497^, ^498^, ^499^, ^500^, ^501^, ^502^, ^503^, ^504^, ^505^
	Cucurbitacin	Mitochondrial ROS; ERK–MTOR–STAT3	Potential anticancer, anti‐inflammatory, antibacterial and antidepressant effects (preclinical)	^506^, ^507^, ^508^
	Quercetin	Modulation of AKT‐MTOR signaling and hypoxia‐induced factor 1α (HIF‐1α) signaling, mitochondria ROS, SIRT1	Antitumor, antioxidant, anti‐inflammatory, antidiabetic, hepatoprotective, and cardioprotective; COVID‐19 treatment (clinical trials)	^509^, ^510^
ROS enhancer	Antimycobacterial antibiotics (isoniazid or pyrazinamide)	Activation of cellular and mitochondrial ROS	Tuberculosis infection (approved)	^511^
BECN1 activator	Tat‐BECN1	A cell‐permeable peptide derived from BECN1; interacting with a negative regulator of autophagy, GAPR‐1	Antivirual, anticancer, cardioprotective, cerebrovascular, and cardiovascular protective, neuroprotective properties (preclinical)	^512^, ^513^, ^514^, ^515^, ^516^, ^517^, ^518^, ^519^, ^520^, ^521^, ^522^, ^523^
	Isorhynchophylline	Dependent on BECN1 function	Neuroprotective properties against neurodegenerative diseases including Parkinson disease (preclinical)	^524^
	BRD1991	Bcl‐2–BECN1 PPI (protein–protein interaction) inhibitors	Experimental agents	^525^
	Xestospongin B	The IP(3)R antagonist inhibit IP(3)R interaction with BECN1	Experimental agents; potential anticancer effects (preclinical)	^526^, ^527^, ^528^, ^529^
Inositol monophosphatase (IMPase) inhibitor	Carbamazepine	Reduction in Ins(1,4,5)P3 and inositol levels; a voltage‐dependent sodium channels blocker	Reduces hepatic fibrosis; fatty liver conditions; neuroprotection (preclinical); treatment of seizures and bipolar disorders (approved)	^530^, ^531^, ^532^, ^533^
	Lithium	Depletion of free inositol, reduction of IP3 levels, and increase in levels of Beclin1; inhibitor of GSK3β.	Bipolar disorder (approved); neurodegenerative disorders, including huntingtin or Parkinson's disease (clinical trials)	^534^, ^535^, ^536^, ^537^, ^538^
TFEB/TFE3 activator	3,4‐Dimethoxychalcone (3,4‐DC)	Stimulating the translocation of TFEB and TFE3 into nuclei;	Cardioprotective effects, and improved the efficacy of anticancer chemotherapy (preclinical)	^539^
	Thiostrepton	Activation of TFEB and TFE3	Stimulate anticancer immune responses of immunogenic cell death (ICD)‐inducing chemotherapeutics (clinical trials)	^540^
HDACI inhibitors	Trichostatin A	FOXO1‐dependent pathways	Anti‐cancer property (clinical trials)	^541^, ^542^, ^543^
	Suberoylanilide hydroxamic acid	FOXO1‐dependent pathways	Cutaneous T‐cell lymphoma treatment (approved); epilepsy, Cushing's disease, breast cancer with a combination with pembrolizumab, tamoxifen (clinical trials)	^541^, ^544^
p38 kinases inhibitor	SB202190	p38alpha/beta kinases inhibitor; TFEB/TFE3‐dependent autophagy and lysosomal biogenesis	Treatment of colorectal cancer (preclinical)	^545^, ^546^
Ca^2+^ channel antagonist	Fluspirilene	Ca^2+^ channel antagonist	Antipsychotic drugs (approved)	^547^, ^548^
	Verapamil	L‐type Ca^2+^ channel antagonist	Nonalcoholic fatty liver, extends lifespan (preclinical); ischemic stroke; type 1 diabetes (clinical trials)	^273^, ^549^, ^550^, ^551^
	Felodipine	Ca^2+^ channel antagonist	Hypertension, cardiovascular diseases (clinical trials); neurodegenerative disorders	^552^
	Loperamide	An opioid receptor agonist that also inhibits voltage‐gated P/Q‐type Ca^2+^ channels; ER stress	Anticancer property (clinical trials)	^551^, ^553^, ^554^
	Amiodarone	Ca^2+^ channel antagonist	Antiarrhythmic agent (approved); hepatitis B virus‐associated hepatocellular carcinoma (preclinical)	^555^, ^556^
Cholesterol depleting agent	MBCD	Enhancing lysosomal V‐ATPase assembly and promoting VAMP3‐mediated autophagosome formation	Experiment agents	^557^, ^558^
Mitophagy inducer	Urolithin A	Reducing mitochondria membrane potential	Antiaging effects via clearance of dysfunctional mitochondria (preclinical)	^559^
Selective autophagy	Autophagy‐targeting chimera (AUTAC)	Hijack autophagy to selectively degrade destines substrate via a standalone tag, S‐Guanylation	Experimental tools	^560^
Unclear mechanism	SMER‐28	Unclear (independent of MTORC1)	Clearance of aggregation‐prone proteins, including amyloid‐β (preclinical)	^561^
	BRD5631	Unclear (independent of MTORC1)	Bacterial clearance; suppresses NPC1‐induced cell death (preclinical)	^562^
	10‐NCP	Unclear (independent of MTORC1)	Against toxicity in a Huntington disease model (preclinical)	^563^
	Alpha‐lipoic acid	Upregulation of autophagy‐related genes	Antiaging effects (as a nutritional supplement), including anti‐inflammatory, cancer prevention, antidiabetic, cardioprotective, and neuroprotective properties, atherosclerosis, hypertension and Alzheimer's disease; nonalcoholic fatty liver disease (clinical trials)	^564^

#### 4.1.1 Activation of AMPK signaling

AMPK, the major cellular energy sensor, responses to the changes of cellular ATP/AMP ratio. Activation of AMPK provides a powerful target to trigger cellular autophagy.^565^ AMPK can be allosterically activated by small molecules through blockage of AMPK T172 dephosphorylation (e.g., A769662).^429^, ^430^ Another direct approach to stimulate AMPK activity is the utilization of AMP mimetics.^566^ For example, 5‐aminoimidazole‐4‐carboxamide ribonucleoside, an adenosine monophosphate, which can be metabolized to an AMP mimetic, is able to cause allosteric activation of AMPK.^431^, ^432^ Indirect activation of AMPK can be achieved via downregulation of the cellular ATP/AMP ratios, which usually resulted from agents blocking ATP production, such as thiazolidinediones, simvastatin and sertraline.^436^, ^437^, ^438^, ^439^, ^440^, ^441^ Both direct and indirect activators of AMPK have been shown to induce autophagy potently and perform therapeutic importance to treat diabetes and related metabolic disorders.^565^

In addition, lifespan extension, or the so‐called anti‐aging function, is one of the most attracting features of AMPK activators. Both AMPK and autophagy are robustly stimulated by CR and excises, which are known strategies to promote health and prolong lifespan in a wide range of animal models with substantial evidences.^567^ The concept of CRMs has been proposed to describe compounds that mimic some of the anti‐aging effects of CR. The key features of CRMs embrace activation of autophagy and global cellular proteins deacetylation.^568^ As a potent approach to activate autophagy, the anti‐aging effects of a number of AMPK activators have been tested and grouped into CRMs, including metformin, resveratrol, aspirin, and spermidine.^434^, ^442^, ^447^, ^482^, ^483^, ^487^ These AMPK‐related CRMs enhance protein deacetylation via induction of an NAD(+)‐dependent deacetylase SIRT1 (sirtuin 1) or inhibition of acetyltransferase EP300 (E1A binding protein p300), which provides additional health benefits in both autophagy‐dependent and ‐independent manners.^434^, ^442^, ^447^, ^482^, ^483^, ^487^

Here, we described a good example to show the function of AMPK activation and the subsequent induction of autophagy: metformin, the first‐line antidiabetic drug, functions as an indirect AMPK activator by suppressing RCC1 (respiratory chain complex‐1) to decrease the cellular ATP/AMP ratio.^433^, ^434^, ^435^ Metformin has also been found to induce autophagy via both AMPK‐dependent and SIRT1‐dependent pathways. Autophagy and SIRT1 have been found to be downregulated in a T2D mouse model.^433^ Therefore, in addition to the energy sensing pathways orchestrated by AMPK, metformin also restores autophagy and SIRT1, which improve diabetic symptoms and contributes to lifespan extension.^433^, ^434^, ^435^ AMPK activation, autophagy induction, and protein deacetylation can be considered as the “trinity” mechanisms efficiently counteracting aging‐associated features, such as cardiovascular, neurodegenerative, malignant, and metabolic diseases. There are still amounts of open questions remained, such as the causal relationship amongst this “trinity,” the criteria to select the appropriate targeting population for different CRMs, and the possible strategies to achieve stronger effects when combining CRMs and other aging‐related signaling regulators.

#### 4.1.2 Inhibition of PIK3CA/AKT/MTOR signaling

PIK3CA/AKT/MTOR signaling is the most‐described inhibitory signaling of autophagy.^569^ Small molecules suppressing the element in this pathway, including AKT, MTOR, and PIK3CA complex, can be used as strong inducers of autophagy. There are mainly two direct strategies to target MTOR activity. First, reagents working as rapamycin, the most famous MTOR inhibitor, allosterically block MTOR kinase activity through destabilizing the MTOR–RAPTOR complex.^193^, ^449^, ^450^, ^451^ Second, small molecules, such as Torin1/2, PP242, and AZD8055, target the ATP binding site on MTOR, hence suppressing its kinase activity in an ATP‐competitive manner.^460^, ^461^, ^462^, ^463^, ^464^, ^465^ In addition to MTOR inhibition, the kinase activities of PI3K or AKT can be allosterically inhibited through small molecules targeting the delta isoform (p110δ) of PI3K or the PH‐domain of AKT, respectively.^476^, ^477^, ^478^, ^479^, ^480^

Suppression of PIK3CA/AKT/MTOR signaling has been applied to the treatments of different types of cancers. Idelalisib (CAL‐101) is the first approved PI3K inhibitor applied in chronic lymphocytic leukemia.^476^, ^477^ Everolimus and temsirolimus, two MTOR inhibitors, have been approved for treatments for chronic myeloid leukemia and prostate cancer patients.^452^, ^453^, ^454^, ^455^ The anticancer efficacies of AZD8055, rapamycin, and rapamycin‐related analogs have been evaluated in a number of Phase I and II clinical trials.^188^, ^193^, ^449^, ^450^, ^451^, ^466^, ^467^, ^468^, ^469^ However, special cautions are needed to interpret the role of autophagy in these anticancer therapies based on suppression of PIK3CA/AKT/MTOR signaling. Autophagy can be used as a mechanism for cancer cells survival under unfavorable microenvironment and enhancement of tumor immune evasion, which largely increases the possibility of drug resistance.^145^, ^570^ Therefore, the synergistic efficacy of the suppression of PIK3CA/AKT/MTOR signaling with autophagy inhibitors has been extensively tested from tumor cell biology to clinical trials, which shows great antitumor potential and needs to be further validated in the future.^571^, ^572^, ^573^, ^574^

#### 4.1.3 Other autophagy activators

Autophagy induction is also druggable through viable targets independent of MTOR signaling, such as BECN1 promotion, HDAC inhibition, TFEB activation, and Ca^2+^ channel antagonist. For instance, 3,4‐DC and thiostrepton stimulate autophagy via activation of TFEB/TFE3, the master transcription factor for lysosome‐related genes and ATGs.^539^, ^540^ These agents have been found to provide additional benefits for the antitumor effects of immunogenic cell death (ICD) inducers, which may indicate the role of autophagy in antitumor immunity.^539^, ^540^

Several Ca^2+^ channel blockers, including fluspirilene, verapamil, loperamide, and amiodarone, have been found to induce autophagy potently.^273^, ^547^, ^548^, ^549^, ^550^, ^551^, ^552^, ^553^, ^554^, ^555^, ^556^ Although the exact mechanisms are not clearly defined, there are reports indicating that the Ca^2+^ channel antagonists‐induced autophagosome formation relates to calpain‐mediated cleavage of ATG5.^273^, ^552^

Inositol monophosphatase (IMPase) inhibitors, including the approved mood‐stabilizing agents, have been reported to perform neuroprotective activity via induction of autophagy. The IMPase inhibitors induce autophagy in a way dependent on decreasing free inositol or myo‐inositol‐1,4,5‐trisphosphate (IP3) levels.^526^, ^527^, ^528^, ^529^ The molecular mechanism underlying the inositol signaling‐regulated autophagy is MTOR independent but is still not fully understood. Studies using an IP3 receptor blockage (Xestospongin B) have demonstrated that IP3 receptor modulates autophagy via its direct interaction with BECN1, which may hint the obligation of BECN1 in IMPase inhibitor‐mediated autophagy.^526^, ^527^, ^528^, ^529^ Interestingly, other BECN1 activators, such as Tat‐BECN1 and Isorhynchophylline, have also been identified to play a neuroprotective role in preclinical studies, which indicates the great potential of BECN1‐depedent autophagy in treatment of neurodegenerative diseases.

### 4.2 Suppression of autophagy

Unlike autophagy activating strategies, which mainly focusing on kinases activity modulation (such as AMPK and MTOR), the strategies inhibiting autophagy usually target the relatively late stages of autophagy, including inhibition of autophagosome formation (both initiation and maturation processes) and lysosomal degradation (Table 3).

**TABLE 3:** Known autophagy pharmacological inhibitors and their potential application in disease

Class	Agent	Mechanism of action	Biological effects and application in disease	References
ULK inhibitor	SBI‐0206965	Pyrimidine analogues inhibiting ULK1 activity in an ATP‐competitive manner; inhibiting AMPK activity in a type IIb inhibitor manner	Potential anticancer effects; diabetic therapy development; traumatic, and neurodegenerative disorders (preclinical)	^52^, ^280^, ^575^, ^576^, ^577^, ^578^, ^579^
	MRT67307 and MRT68921	Small molecule inhibitors (also known as TBK1 inhibitor)	Potential anticancer effects (preclinical)	^579^, ^580^
	ULK1‐101	A small molecule inhibitor	Sensitizes KRAS mutant lung cancer cells to nutrient stress (preclinical)	^581^
PI3K class III complex inhibitor	3‐MA	A dual inhibitor of PI3K (class I and III)	Experimental tool	^582^, ^583^, ^584^, ^585^, ^586^
	Wortmannin	The sterol‐like fungal metabolite inhibiting PI3K (class I and III) reversibly	Experimental tool	^582^, ^587^, ^588^, ^589^
	SAR405	Inhibiting the ATP‐binding cleft of PIK3 class III (with higher selectivity when compared with wortmannin and 3‐MA)	Enhancing the antitumor efficacy of cisplatin or mTOR inhibitors; improves anti‐PD‐1/PD‐L1 immunotherapy; promotes host cell death during latency reversal of HIV (preclinical)	^590^, ^591^, ^592^, ^593^, ^594^, ^595^
	SB02024	Selectively inhibiting PIK3C3/VPS34	Improves the sensitivity of breast cancer cells to Sunitinib; improves anti‐PD‐1/PD‐L1 immunotherapy (preclinical)	^595^, ^596^
	VPS34‐IN1	Selective cell permeable PIK3C3/VPS34 inhibitor in nanomolar level	Anticancer effects including acute myeloid leukemia and CNS tumor; SARS‐CoV‐2 inhibitors; (preclinical)	^319^, ^597^, ^598^
	LY294002	A reversible inhibitor of PI3K	Promoting antitumor effect of different chemotherapy drugs (preclinical)	^587^, ^599^, ^600^
	PIK‐III	Inhibiting PIK3C3/VPS34 activity via binding to a unique hydrophobic pocket (with higher selectivity when compared with wortmannin and 3‐MA)	Treatment for CML patients; SARS‐CoV‐2 inhibition (preclinical)	^319^, ^594^, ^601^
	Autophinib	Inhibiting PIK3C3/VPS34 in an ATP‐competitive manner	Potential anticancer effects in cell level (preclinical)	^602^, ^603^, ^604^, ^605^
	Azaquindoles	Inhibiting PIK3C3/VPS34 in an ATP‐competitive manner	Experimental agents	^606^
	Spautin‐1	Targeting the BECN1 subunit of PIK3C3/VPS34 complexes via Inhibition of USP10 and USP13	Anticancer effects (preclinical)	^607^, ^608^, ^609^
	Compound 19a	Inhibiting the Beclin 1‐ATG14L PPI, which is required for PI3KC3‐C1 formation	Experimental agents	^610^
ATG4B inhibitor	NSC185058	ATG4B antagonist	Enhancing the antitumor effects of radiotherapy or temozolomide (TMZ) in glioma treatment (preclinical)	^611^, ^612^, ^613^
	FMK‐9a	Suppressing ATG4B through formation of a covalent bond with Cys74	Experimental agents	^614^, ^615^
PP2A inhibitor	Okadaic acid	Enhancing phosphorylated ATG4B (Ser316) via inhibition of PP2A; Inducing cytokeratin cytoskeleton disruption	Experimental agents	^616^, ^617^, ^618^
ATG7 inhibitor	Pyrazolopyrimidine sulfamate compounds	Targeting ATG7 and covalently binding to ATG3	Experimental agents	^619^
STX17 inhibitor	EACC	Suppressing the translocation of STX17 on autophagosomes	Experimental agents	^620^, ^621^, ^622^
LC3 inhibitor	S130	Blocking the recycling of LC3‐I via inhibiting the delipidation of LC3‐II	Potential anticancer effects (preclinical)	^623^
GRAMD1A inhibitor	Autogramins	Disturbing the cholesterol distribution via competing with cholesterol binding to the GRAMD1A, which is required for autophagosome initiation	Experimental agents	^624^
Cytoskeletal protein inhibitor	CA‐5f	Inhibiting autophagy–lysosome fusion via inhibiting cytoskeletal proteins and membrane traffic proteins	A novel late‐stage autophagy inhibitor with potential clinical application for NSCLC therapy (preclinical)	^625^
	Vinblastine	Destabilizing microtubule function to suppress autophagosome maturation	Treatment on AML and recurrent solid tumor with sirolimus (clinical trials)	^626^, ^627^, ^628^
Small GTPase modulating agent	Thapsigargin	Inhibiting recruitment of RAB7‐mediated autophagosome–lysosome fusion	Thasigargin based prodrugs mipsagargin is in Phase II clinical testing for solid tumor (clinical trials)	^629^, ^630^
	Vacuolin‐1	Inhibiting RAB5A‐mediated autophagosome–lysosome fusion; PIKfyve ihibitor	Potential anti‐COVID‐19 effects; inhibition of migration and metastasis of cancer cells (preclinical)	^631^, ^632^, ^633^, ^634^, ^635^
Lysosomotropic agent	CQ and HCQ	Blocking lysosomal acidification	Antimalarial agent (approved); anticancer effects, alone or combined with other chemotherapy drugs (clinical trials); antiviral effects including COVID‐19 (clinical trials)	^306^, ^319^, ^558^, ^594^, ^636^, ^637^, ^638^, ^639^
	Lys05	A dimeric form of CQ	Treatment for CML patients (preclinical)	^594^, ^640^
	DQ661	A dimeric quinacrine	Potential anti‐cancer effects (preclinical)	^641^
	DC661	A dimeric CQ	Potential anticancer effects (preclinical)	^642^
	GNS561	Inhibiting PPT1 function, which results in lysosomal Zn2+ accumulation and blockage of cathepsin enzyme activity	Liver cancers treatment (clinical trials); COVID‐19 treatment (clinical trials)	^643^, ^644^, ^645^
	ARN5187	A lysosomotropic REV–ERBβ ligand	Potential anticancer effects	^646^
	ROC‐325	A compound with structural motifs of both HCQ and lucanthone	Superior preclinical anticancer activity; augments the antileukemic activity of azacitidine	^647^, ^648^
	Azithromycin	Blocking lysosomal acidification	With poorly understood anti‐inflammatory properties; potential antitumor effects	^649^, ^650^, ^651^
	Autoquin	Blocking lysosomal acidification	Experimental agent	^652^
V‐ATPase inhibitors	Bafilomycin A1	Inhibiting V‐ATPase by blocking the dissociated V(1)‐ATPase; suppression Ca‐P60A/SERCA‐dependent autophagosome–lysosome fusion	Experimental agent	^558^, ^637^, ^653^
	Concanamycin A	Highly potent V‐ATPase inhibitors via binding to V(0) subunit c	Antiviral effects, including HIV, influenza (preclinical)	^654^, ^655^, ^656^
	Verucopeptin	Interacting with ATP6V1G to block V‐ATPase activity	Potential therapeutics against multidrug‐resistant cancers (preclinical)	^657^
	Salicylihalamide A	Blocking the ATPase activity by inhibiting the V(0) domain	Experimental agents	^658^
	BRD1240	Suppresses V‐ATPase function	Experimental agents	^659^
Lysosome function inhibitor	Lucanthone (Miracil D)	Inducing lysosomal membrane permeabilization (unclear molecular mechanism)	Antischistosomal drug (approved); potential anticancer effect (clinical trials for brain tumor and brain metastases)	^660^, ^661^
PIKfyve inhibitor	Apilimod	Disrupts lysosomal homeostasis and intracellular trafficking; Inducing secretory autophagy	Treatment of non‐Hodgkin lymphoma (clinical trials); treatment of COVID‐19 (clinical trials)	^662^, ^663^, ^664^
TRPML1 inhibitor	ML‐SI1	Small molecule inhibits Mucolipin TRP channel 1 (TRPML1) as the key lysosomal Ca^2+^ channel	Experimental agent	^665^
Na(+),K(+)‐ATPase inhibitor	Cardiac glycosides	Antagonists of Na(+),K(+)‐ATPase, inhibit autotic cell death	Extensively used in the past for treatment of heart failure and arrhythmia treatment inhibited autosis death, which might occur in liver and heart of patients with severe anorexia nervosa clinical potential of CGs in anticancer and other therapies	^523^, ^666^, ^667^, ^668^
Mitophagy inhibitor	Mdivi‐1	A pharmacological inhibitor of DRP1, inhibit mitochondria fission	Chronic obstructive pulmonary disease (COPD); pressure overload‐induced heart failure; type 2 diabetes	^669^, ^670^, ^671^
Unclear mechanism	Oxautin‐1	Inhibiting both autophagosome biogenesis and autophagosome maturation	Experiment agents	^672^
	Clomipramine	Blocking autophagolysosomal fluxes	Treatment of psychiatric disorders (approved); Potential anticancer effects (preclinical)	^626^, ^673^
	Verteporfin	Disturbing the shape of double membrane autophagosome structure	A benzoporphyrin derivative used in photodynamic therapy (approved)	^674^, ^675^

#### 4.2.1 Inhibition of autophagy initiation and autophagosome formation

Small molecules and natural products targeting ULK1 complex and PI3KC3–C1 are two major approaches to suppress autophagy initiation, which results in the blockage of autophagosome biogenesis. These reagents have been reported to have great potentials in anticancer, antidiabetic therapy, and treatment of neurodegenerative disorders. However, special attention should be taken since these kinase inhibitors are commonly with a broad inhibition of kinase activity, which may bring undesired impacts or even results opposing to its original intention.

The early identified ULK inhibitors, MRT67307 and MRT68921, have been screened from a closely related series of analogues generated during the original TBK1 (TANK‐binding kinase 1) screen, hence the side effects of TBK1 inhibition are unavoidable for these drugs.^580^ Another early identified ULK inhibitor SBI‐0206965 also demonstrates a potent AMPK inhibitory effect.^576^, ^577^ The recently reported ULK‐101 is considered as the most promising ULK inhibitor with a significantly higher potency and selectivity when comparing to the previous inhibitors, while further investigation in vivo is required to proceed with preclinical testing.^581^

3‐MA, a pan‐PI3K inhibitor widely used as a useful experiment tool to block early stage of autophagy, has been found to block the autophagy‐promoting PI3K complex (class III) transiently, while inhibits the autophagy‐suppressing PI3K complex (class I) persistently. In this scenario, 3‐MA plays a time‐dependently dual role in autophagy modulation. The similar problem of poor selectivity is also unavoidable when using another PI3K inhibitor, wortmannin.^582^ Therefore, to avoid the side effects caused by PIK3CA inhibition, small molecules with higher specificity for PIK3C3 are screened and selected, such as SAR405, VPS34‐IN1, PIK‐III, and SB02024.^319^, ^590^, ^591^, ^592^, ^593^, ^594^, ^595^, ^596^, ^597^, ^598^, ^601^ These reagents have been found to inhibit autophagy and sensitize cancer response to immunotherapy (anti‐PD‐1/PD‐L1) or chemotherapy (sunitinib and cisplatin) in preclinical studies.^595^, ^596^ However, PIK3C3 targeting strategy is still not perfect for specific autophagy inhibition, since PIK3C3 is the key component for PI3KC3–C1 for autophagy initiation and PI3KC3–C2 for endosomal trafficking.^676^ Suppression of PIK3C3 also alters endosomal pathways in addition to autophagy inhibition. Recently, compound 19a has been screened and studied as a small molecule, which only disturbs the interaction between BECN1 and ATG14 without interference with PI3KC3–C2.^610^ This study demonstrates the viability of targeting protein–protein interaction (PPI) to control cellular autophagy process.

In addition to these kinases targeting approaches, diverse novel targets specified for autophagosome formation have been developed, including inhibition of ATG4B, ATG7, and alteration of autophagosome lipid composition by inhibiting lipid transport proteins.^611^, ^612^, ^613^, ^614^, ^615^, ^619^, ^624^ Although further validation of the potency and selectivity are still needed, these tools provide great opportunities to observe the detailed modulating processes on autophagosome formation depending on ATG4B, ATG7, or lipid transportation.

#### 4.2.2 Inhibition of autophagosome maturation and lysosome fusion

The enclosing process of double membrane autophagosomes and their fusion with lysosomes can be targeted for autophagy inhibition. Agents blocking cytoskeleton and membrane transport proteins, such as CA‐5f and vinblastine, destabilize cellular organelles (including autophagosomes and lysosomes) movements, resulting in autophagy inhibition via the disturbance of autophagosome–lysosome fusion.^625^, ^626^, ^627^, ^628^ However, these agents are usually with unwanted side effects and unavoidable cytotoxicity because of the broad impacts caused by cytoskeleton inhibition. An alternative approach to block autophagosome formation is to modulate the activity of small GTPases required for the autophagosomes and lysosomes membrane fusion, but also with the problem of strong side effects. For example, thapsigargin, blocking agent for RAB7 recruitment, is used to be considered as an inhibitor for autophagosome fusion. However, it also induces ER stress and inhibits SERCA pump.^629^, ^630^ Vacuolin‐1, which activates RAB5A GTPase to block autophagosome–lysosome fusion, also causes strong side effects via suppressing protein sorting and endocytic pathways simultaneously.^631^, ^632^, ^633^, ^634^, ^635^

More specific strategies targeting autophagosome maturation are proposed and studied recently. EACC blocks the recruitment of STX17 on autophagosome membranes, which provide an interesting tool to understand the specific function of STX17 on autophagosome formation.^620^, ^621^, ^622^ In addition, S130 has been reported to decrease LC3‐II delipidation and hence suppresses the recycling of LC3, which has been validated via in vivo model and shows inhibitory effects on colon cancer cells.^623^ Vertepofin, a US FDA‐approved drug that sensitizes pancreas cancer patients to gemcitabine treatment, inhibits autophagosome maturation via disturbance of double membrane structure, possibly via its interaction with LIR motifs.^674^, ^675^

#### 4.2.3 Suppression of lysosome activity

Autophagy is known as a one‐way ticket to lysosome degradation. Suppression of lysosome activity significantly blocks autophagic degradation. Here, we described the main lysosomal targeting strategies.

Lysosomotropic drugs are the weak bases compounds incorporated and accumulated into cellular acidic organelles (lysosomes and late endosomes), leading to increased acidic pH value, destabilization of lysosomal membranes, and subsequent blockade of lysosomal functions.^677^ CQ and HCQ, as the most‐studied lysosomotropic agents, are widely used as traditional antimalaria drugs, and draw more and more attentions because of its possible therapeutic anti‐COVID‐19 efficacy.^306^ The rationale to combine autophagy inhibitors with chemo‐, radio‐, or immune‐antitumor therapy has been proposed based on the importance of autophagy in tumor resistance. As the only autophagy inhibitory drugs approved by US FDA, CQ spurred multiple preclinical studies and clinical trials to investigate their anticancer efficacies alone or in combination with other anticancer treatments. However, although CQ/HCQ have been proved to be safely tolerated in a relatively high dosage and preliminarily effective in a subset of patients (such as patients with melanoma, colon cancer, and kidney cancer),^678^ there are still a number of open questions left: (1) the suitable in vivo autophagic marker to confirm whether autophagy is fully blocked by CQ/HCQ; (2) the criteria to select the sensitive population of CQ; (3) side effects including cardiomyopathy. Derivatives modified based on the structure of CQ and other antimalaria drugs have been systematically explored to optimize the potency on autophagy inhibition. Further profiling is needed for this new generation of lysosomotropic agents, including Lys05, DQ661, DC661, lucanthone, and ROC‐325.^594^, ^640^, ^641^, ^642^, ^647^, ^648^, ^660^, ^661^

V‐ATPase inhibitors prevent lysosome acidification via blockage of lysosomal proton pump activity directly. Multiple nature products, including bafilomycin A1, concanamycin A, and salicylihalamide A, are screened and identified as V‐ATPase inhibitors to suppress autophagic degradation with high potency.^558^, ^637^, ^653^, ^654^, ^657^, ^658^ The lysosomal located V‐ATPase are also required for nutrient sensing and MTORC1 activation. These features may provide additional advantages of V‐ATPase inhibitors in cancer treatment. For instance, a novel identified V‐ATPase inhibitor, verucopeptin, leads to the disassociation of MTOR from lysosome membrane, hence suppressing the MTORC1 activity in addition to autophagy degradation. Verucopeptin has been shown to effectively prevent multidrug resistance and cancer progression in vivo.^657^

In addition to the lysosomotropic and V‐ATPase inhibiting strategies, there are multiple mechanistic targets to antagonize lysosome proteolysis, such as the key lysosomal Ca^2+^ channel TRPML1 (mucolipin TRP channel 1) and lysosomal membrane permeabilization.^649^, ^650^, ^651^, ^665^

Several lysosome function inhibitors, including CQ and bafilomycin A1, routinely serve as useful tools to investigate autophagic flux and study the function of autophagy in different physiological or pathological situations. However, it should be cautious when using the lysosomal inhibitors in autophagy studies. These reagents also disturb other lysosome involving cellular processes in addition to autophagic degradation, including endocytosis, phagocytosis, and nutrient sensing. Multiple approaches and carefully designed controls are needed to determinate autophagic flux, especially when the lysosome‐related processes are also involved.

## 5 SUMMARY AND PERSPECTIVES

The modern era of autophagy research started more than 70 years ago when Christian de Duve discovered lysosome in 1950s and coined the term autophagy in 1960s, while the real breakthrough came only when the ATGs were identified in yeast by Yoshinori Ohsumi in 1990s. In the past near 3 decades, we have observed tremendous progresses in the autophagy field, from the molecular mechanisms, biological functions, and implications in health and diseases. The autophagy research covers almost all the model organisms, from plant, yeast, C. elegans, all the way to mouse and human. In particular, the availability of various transgenic animal models in which ATGs and related genes are manipulated have provided valuable resources for deeper understanding of autophagy and its association with human diseases. More importantly, the autophagy research has moved from basic mechanistic research to translational studies, with numerous autophagy inhibitors or activators being in preclinical and clinical experiments, and we have attempted to summarize all the above points to showcase the importance of autophagy.

However, despite the remarkable achievements, there are equally important challenges and bottlenecks in autophagy research.^6^ First, in comparison with macroautophagy, which is autophagosome dependent, relatively little is known about microautophagy in which various cargos are delivered to lysosome for degradation independent of the canonical autophagy machinery and autophagosome formation.^10^ For example, how many distinct types of microautophagy exist in eukaryotes? What are the molecular mechanisms unique to microautophagy? Are there any specific proteins that mediate the invagination of autophagic cargos into the lysosome? What factors that trigger microautophagy instead of macroautophagy? How do microautophagy and macroautophagy coordinate with each other to maintain the cellular homeostasis? Answering these questions may help to expand the scope of autophagy research and provide new insights into the biological functions and importance of microautophagy in diseases.

Second, there is increasing appreciation to the importance of selective autophagy. At present, a variety of selective autophagy have been identified, from mitophagy (mitochondria), ER‐phagy (ER), aggrephagy (protein aggregates), lipophagy (lipid droplets), lysophagy (lysosome), glycophagy (glycogen), ferritinophagy (ferritin), just to name a few.^679^ These selective forms of autophagy are often featured in specific type of cells/tissues, induced by specific stimuli and mediated by specific adaptors, thus with particularly relevance to certain type of diseases. One good example is mitophagy and its close implication in neurodegenerative disorders such as PD.^236^, ^680^ Obviously, much more work is needed to understand these forms of selective autophagy, from molecular mechanisms to functional implications in health and disease.

Third, only limited number of transgenic animal models are available that mimic development of autophagy‐related disease via either deletion or overexpression of ATGs. Such animal models are indeed important for understanding the exact role of autophagy in disease, as well as for development of autophagy‐targeted therapeutics. In addition to mouse models, establishment of other model organisms such as zebrafish, drosophila and *C. elegans* will also be useful.

Fourth, the autophagy research field still faces one technical challenge: lack of proper tools for measuring autophagic flux in vivo, especially in human specimen. Such tools are actually critical for translating the knowledge of autophagy from bench to bed, as we need to know whether autophagy activators or inhibitors should be applied to patients with either impaired or activated autophagy under specific disease conditions.

Last, despite the tremendous efforts in developing autophagy‐specific therapeutic agents, most of them are still at the earlier stage of development, mainly preclinical. The only autophagy inhibitor in clinical trials is the repurposed antimalaria drug CQ/HCQ based on their lysosomotropic property and inhibitory effect on lysosomal function.^344^, ^681^ It is expected that more such autophagy‐targeted agents, both for macroautophagy and microautophagy, nonselective and selective autophagy, will be entering the clinical stage and bring real benefits to patients.

## CONFLICTS OF INTERESTS

Canhua Huang is an editorial board member of MedComm. The paper was handled by another Editor and has undergone a rigorous peer‐review process. Author Canhua Huang was not involved in the journal's review of/or decisions related to this manuscript. The other authors have no conflicts of interest to declare.

## ETHICS STATEMENT

Not applicable.

## AUTHOR CONTRIBUTIONS

H. M. S., C. W., and W. H. conceived the structure of the manuscript. G. L., Y. W., Y. S., and H. M. S. drafted initial manuscript, with contribution from C. W., W. H., C. H. H. H. M. S., G. L., Y. S., Y. W., Z. Z., C. W., W. H., and C. H. H. revised the manuscript. G. L., Y. W., Y. S., and Z. Z. prepared the figures. All authors read and approved the final manuscript. G. L., Y. W., and Y. S. contributed equally to this study.

## Data Availability

Not applicable.
